# Age- and sex-specific reference values for phosphate homeostasis parameters—fibroblast growth factor 23 and soluble Klotho

**DOI:** 10.1093/ckj/sfag147

**Published:** 2026-05-08

**Authors:** Katharina Schermuly, Hannah Weber, Anna Tschirner, Thomas Rebe, Dieter Haffner, Maren Leifheit-Nestler

**Affiliations:** Department of Pediatric Kidney, Liver, Metabolic and Neurological Diseases, Hannover Medical School, Hannover, Germany; Department of Pediatric Kidney, Liver, Metabolic and Neurological Diseases, Hannover Medical School, Hannover, Germany; Department of Pediatric Kidney, Liver, Metabolic and Neurological Diseases, Hannover Medical School, Hannover, Germany; Department of Occupational Medicine, Hannover Medical School, Hannover, Germany; Department of Pediatric Kidney, Liver, Metabolic and Neurological Diseases, Hannover Medical School, Hannover, Germany; Department of Pediatric Kidney, Liver, Metabolic and Neurological Diseases, Hannover Medical School, Hannover, Germany

**Keywords:** FGF23, phosphate, soluble Klotho, TmP/GFR, urinary calcium-to-creatinine ratio

## Abstract

**Background:**

Currently, uniform reference values for parameters of phosphate (Pi) homeostasis—the phosphaturic hormone fibroblast growth factor 23 (FGF23) and its coreceptor, soluble Klotho (sKlotho)—are used for women and men regardless of age. This may not be adequate, especially in older subjects. We established Lambda–Mu–Sigma (LMS)-based continuous age- and sex-specific reference values for key parameters of Pi homeostasis, FGF23 and sKlotho.

**Methods:**

The HAnnover Reference values for Adults study included 504 participants (51% women) aged 18–70 years. Main outcome measures were serum Pi, intact (iFGF23) and total FGF23, sKlotho, tubular maximum phosphate reabsorption per glomerular filtration rate (TmP/GFR), fractional tubular reabsorption of phosphate (TRP), and urinary calcium-to-creatinine (Ca/Crea) and phosphate-to-creatinine ratios.

**Results:**

All parameters examined showed statistically significant differences between the sexes. There was a constant decrease in renal Pi reabsorption capacity (TmP/GFR, TRP) and serum Pi concentrations in older men, beginning around their sixth decade of life, whereas the parameters of Pi homeostasis in women hardly changed with age. Women had significantly lower iFGF23 but slightly higher total FGF23 concentrations. sKlotho concentrations decreased with age in both sexes, with women generally having higher concentrations than men regardless of the age-related decline in kidney function. Women taking estrogen-containing oral contraceptives had lower sKlotho concentrations than their peers. Women showed higher urine Ca/Crea values than men.

**Conclusion:**

The presented LMS-based continuous reference values for key parameters of Pi homeostasis enable the calculation of standardized *z*-scores to facilitate test result interpretation in women and men.

## Graphical Abstract

**Graphical Abstract:**
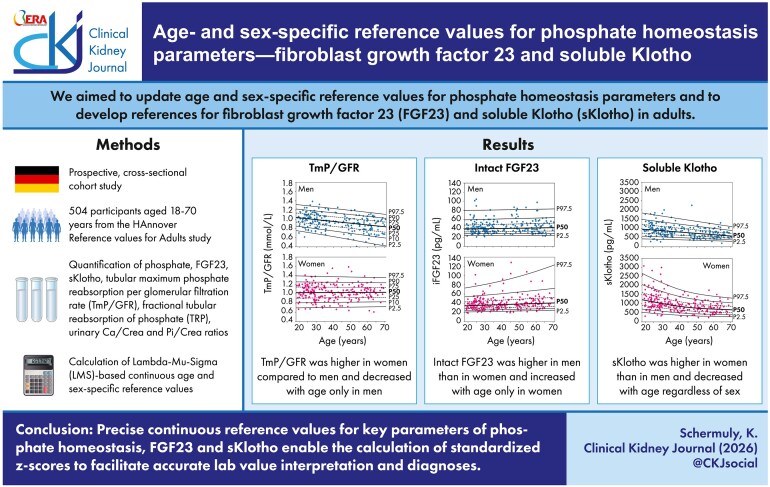

KEY LEARNING POINTS **What was known:**

- Currently, uniform reference values for parameters of phosphate (Pi) homeostasis—the phosphaturic hormone fibroblast growth factor 23 (FGF23) and its coreceptor, soluble Klotho (sKlotho)—are used for women and men regardless of age.
- Preliminary studies suggest that serum Pi values differ between women and men, particularly in older age groups, which calls into question the diagnostic approach to renal Pi wasting disorders using uniform reference values.

**This study adds:**

- We established Lambda–Mu–Sigma (LMS)-based continuous age- and sex-specific reference values for key parameters of Pi homeostasis including serum Pi, tubular maximum phosphate reabsorption per glomerular filtration rate (TmP/GFR) and fractional tubular reabsorption of phosphate (TRP), total FGF23, intact FGF23 (iFGF23), and sKlotho for the age range from 18 to 70 years.
- All parameters examined showed statistically significant differences between the sexes. There was a constant decrease in renal Pi reabsorption capacity (TmP/GFR, TRP) and serum Pi concentrations in older men, beginning around their sixth decade of life, whereas the parameters of Pi homeostasis in women hardly changed with age.
- Women had significantly lower intact (iFGF23) but slightly higher total FGF23 concentrations. Women generally had higher concentrations of sKlotho than men regardless of the age-related decline in kidney function. Women taking estrogen-containing oral contraceptives had lower sKlotho concentrations than their peers.

**Potential impact:**

- The precise LMS-based continuous reference values provided for the key parameters of Pi homeostasis, sKlotho and FGF23, enable the calculation of standardized patient *z*-scores.
- This facilitates the interpretation of test results in clinical practice and in studies and enables the correct diagnosis of disorders of renal Pi handling in women and men.

## INTRODUCTION

Phosphate (Pi) homeostasis is maintained by the kidneys and intestine to allow adequate mineralization of bone and teeth [1]. Renal Pi handling is mainly controlled by the phosphaturic hormones parathyroid hormone and fibroblast growth factor 23 (FGF23), both of which downregulate the sodium-dependent Pi cotransporters NPT2a and NPT2c in the proximal tubule. Laboratory parameters of Pi homeostasis used in the clinical work-up in patients with hypophosphatemia include serum Pi, and measures for renal Pi wasting, e.g. the ratio of the tubular maximum reabsorption of phosphate to the glomerular filtration rate (TmP/GFR), tubular reabsorption of phosphate (TRP), and the urinary phosphate-to-creatinine ratio (Pi/Crea). Determination of FGF23 levels is used to differentiate between FGF23-mediated hypophosphatemia, e.g. due to X-linked hypophosphatemia, and other causes of renal hypophosphatemia, such as Fanconi syndrome or genetic defects in NPT2a and NPT2c [2, 3]. Finally, the circulating levels of the soluble form of the anti-aging hormone Klotho (sKlotho), which acts as an essential FGF23 coreceptor in the kidney, were shown to be associated with kidney function and all-cause mortality in adult patients with chronic kidney disease (CKD), but data are inconsistent [4, 5].

Currently, uniform reference values for parameters of Pi homeostasis—FGF23 and sKlotho—are used for women and men regardless of age, which may not be adequate, especially in older subjects [6–8]. In addition, intake of estrogen-containing oral contraception (OC) in women may also impact parameters of Pi homeostasis by modifying the expression of the FGF23 coreceptor Klotho in the kidney [9, 10].

The development of Lambda–Mu–sigma (LMS)-based continuous reference values for key laboratory values of Pi homeostasis improved data interpretation in clinical practice in children as it accounts for the physiological differences between boys and girls and changes during maturation and allows calculation of standardized patient *z*-scores [11, 12]. Therefore, we developed sex-specific and, where appropriate, age- specific LMS-based continuous reference percentiles for key parameters of Pi homeostasis in the HAnnover Reference values for Adults (HARA) study covering the ages of 18 to 70 years. These included serum Pi, TmP/GFR and TRP, urine Pi/Crea and calcium-to-creatinine ratio (Ca/Crea), total FGF23 and its biologically active form, intact FGF23 (iFGF23), and sKlotho.

## MATERIALS AND METHODS

### Participants and study design

In 2024, we initiated the HARA study, which aims to establish sex-specific reference values for relevant laboratory parameters in adults. This prospective, single-center cross-sectional study included volunteers aged 18–70 years who underwent occupational health and/or legally recommended preventive medical check-ups at Hannover Medical School or outpatient clinics in Hannover (56%), as well as volunteers from the general population (44%). Exclusion criteria for this study were: any form of growth or skeletal disorders, a fracture in the last three months, infections, inflammatory disease, C-reactive protein (CRP) >10 mg/l, malnutrition, anemia (hemoglobin: men <13 g/dl, women <12 g/dl), history of impaired cardiac function or liver diseases, proteinuria (urinary protein-to-creatinine ratio >0.3 g/g) [13], pregnancy, a history of cancer in the last 10 years, medication potentially impairing bone and mineral metabolism, or a reduced (<5th percentile) age-related estimated glomerular filtration rate (eGFR) [14]. A total of 525 subjects were screened for enrollment between March 2024 and December 2024, of whom 504 (51% female) were included in this study (Table 1). The vast majority of participants were Caucasian (92.9%), and 5.5% and 0.7% were of Asian and Black African ethnic background, respectively. Blood and urine samples were collected between 8 a.m. and 2 p.m., irrespective of the fasting state. Time to centrifugation was <2 h. Ethylenediaminetetraacetic acid (EDTA) plasma, serum, and urine samples were aliquoted, stored at −80°C, and further analysed within 1 year with a maximum of one freeze–thaw cylcle. This study was performed in compliance with the Declaration of Helsinki and was approved by the Ethics Committee of the Hannover Medical School (#11127_BO_S_2023). All participants provided written informed consent prior to enrollment.

**Table 1:** Demographic, anthropometric, biochemical, and lifestyle parameters of the HARA cohort.

	All	Men	Women	
Variables	(*n* = 504)	(*n* = 246)	(*n* = 258)	*P*-value
Age, years	36.5 (27.1; 54.2)	36.0 (27.8; 57.5)	36.7 (27.0; 51.2)	.105
Height, cm	175 (168; 182)	182 (176; 186)	168 (164; 172)	< .001
Body weight, kg	75.0 (64.0; 86.0)	83.0 (75.0; 92.0)	65.0 (59.8; 74.0)	< .001
BMI, kg/m^2^	24.0 (21.0; 26.0)	25.0 (23.0; 27.0)	22.0 (20.8; 25.0)	< .001
S-Crea, µmol/l	79 (70; 90)	88 (81; 97)	71 (64; 78)	< .001
eGFR, ml/min/1.73 m^2^	88 (78; 100)	88 (79; 101)	88 (77; 99)	.5
UACR, mg/g crea	5.5 (3.8; 11.2)	4.3 (3.0; 8.7)	6.8 (4.2; 12.1)	< .001
Smoker, *n* (%)	73 (14.7%)	28 (15.6%)	35 (13.9%)	.596
Estrogen intake, *n* (%)	n.a.	n.a.	68 (26%)	n.a.
Physical activity, *n* (%)				
<30 min/week	84 (17.1%)	35 (14.6%)	49 (19.5%)	.151
30–60 min/week	116 (23.6%)	42 (17.5%)	74 (29.5%)	.002
60–120 min/week	133 (27.1%)	70 (29.2%)	63 (25.1%)	.304
>120 min/week	158 (32.2%)	93 (38.8%)	65 (25.9%)	.002

Data are presented as median (IQR) or percentage (%); *P*-values were calculated using Mann–Whitney U test or chi-squared test, respectively. S, serum; Crea, creatinine; n.a., not applicable.

### Investigations and biomarker analysis

All participants completed a questionnaire covering age, sex, height, weight, smoking habits, weekly exercise volume, and intake of OC or estrogen-containing oral medication (OE) for the treatment of postmenopausal symptoms in women. Body mass index (BMI) was calculated as follows: BMI = body weight (kg)/height (m) squared. Serum calcium, CRP, Pi, aspartate aminotransferase, creatine kinase, creatinine, and cystatin C, and urinary Ca, Pi, protein, albumin, glucose, and creatinine concentrations were analysed employing routinely used automated methods (Cobas 8000, Roche Diagnostics, module c701, Mannheim Germany). The European Kidney Function Consortium equation, based on serum creatinine, was applied to determine eGFR [15]. TRP and TmP/GFR were calculated using the following equations: TRP = 1 − [(UP/SP) × (SCr/UCr)]; and TmP/GFR = SP − (UP/UCr) × SCr, where UP = urinary Pi, SP = serum Pi, SCr = serum creatinine, and UCr = urinary creatinine [16, 17]. Albuminuria was graded as A1 (normal or mild increase): urine albumin-to-creatinine ratio (UACR) <30 mg/g, A2 (moderate increase): UACR between 30 and 300 mg/g, and A3 (severe increase): UACR >300 mg/g, in accordance with the definition of the Kidney Disease: Improving Global Outcomes 2024 guidelines [13]. FGF23 concentrations were determined in EDTA plasma samples using two different commercially available enzyme-linked immunosorbent assay (ELISA) kits. The C-terminal ELISA quantifies total FGF23, detecting the intact hormone and its C-terminal fragments. In contrast, the iFGF23 assay is specific for the biologically active, uncleaved form of the hormone [18]. ELISA kits were employed to quantitatively assess plasma levels of iFGF23 (Immutopics, Catalog # 60-6600, RRID: AB_2891250) and C-terminal FGF23 (Immutopics, Catalog # 60-6100, RRID: AB_2722648), and serum levels of sKlotho (Immuno Biological Laboratories, Catalog # 27998, RRID: AB_2750859). The assays were conducted in accordance with the manufacturer’s instructions. Each sample was measured in duplicate using the Tecan Infinite M200 Pro, with quantitative evaluation performed through Magellan 7.2 software. The inter- and intra-assay coefficients of variation were 11.4% and <5% for sKlotho, <5% for total FGF23, and 9.1% and <5% for iFGF23, respectively.

### Statistical analysis

Descriptive statistics are presented as median (interquartile range; IQR) for non-normally distributed data (determined by the Shapiro–Wilk test) or as a percentage. Differences between groups were evaluated using either the Mann–Whitney U test or the chi-squared test. Associations between variables were assessed through univariate and multiple stepwise linear regression analyses. Statistical significance was defined as *P* < .05. All statistical procedures were carried out using SPSS software, version 29.0 (IBM Corporation, New York, USA) and GraphPad Prism, version 10.4.2. Percentile reference curves, specific for sex and, where applicable, specific for age, were created using the LMS method in RefCurv 0.4.2 (Windows), employing generalized additive models for location, scale, and shape [11]. For age-independent parameters, namely total FGF23 and urine Ca/Crea, we created violin plots. The LMS procedure characterizes the data distribution through three key parameters: Lambda, reflecting skewness; Mu, representing the median; and Sigma, indicating the coefficient of variation [19]. Data points with 2 SD above or below the 97.5th and 2.5th percentile, respectively, were identified as outliers and removed in the process of constructing the respective percentile curve and violine plots. To do so, we had to exclude none for Ca/Crea men and women and sKlotho men, one each for Pi men and women, iFGF23 women, and sKlotho women, two for iFGF23 men, and four each for total FGF23 men and women. Consequently, these participants were also excluded for all calculated ratios.

## RESULTS

### Participants

A total of 504 participants (258 female) fulfilled the criteria for this reference study (Table 1). Their median age was 36.5 years (IQR 27.1; 54.2). The values for height, weight, BMI, serum creatinine, and eGFR were within the normal range, with higher median values in males compared to females (each *P* < .001), with the exception of eGFR. About 15% of participants were smoker. A2 albuminuria was detected in 18/476 (3.8%) participants, with slightly higher median UACR in females compared to males (*P* < .001). None of the participants had A3 albuminuria. Twenty-six percent of female participants reported taking estrogens, including OC and OE in 24% and 2% of women, respectively. Physical activity differed between men and women, with a higher percentage of men being active for more than 120 min per week, while women were more often active for between 30 and 60 min per week.

All parameters of Pi homeostasis examined differed statistically between the sexes. In addition, all parameters except total FGF23 and urine Ca/Crea were associated with age in men and/or women. We provide sex-specific reference percentiles and sex-specific violin plots for age-dependent and age-independent parameters. The sex and/or age-specific LMS values and normal ranges for the laboratory parameters are given in Supplementary Tables S1–S10.

### LMS percentiles for serum Pi, TmP/GFR, and TRP

**Figure 1:**
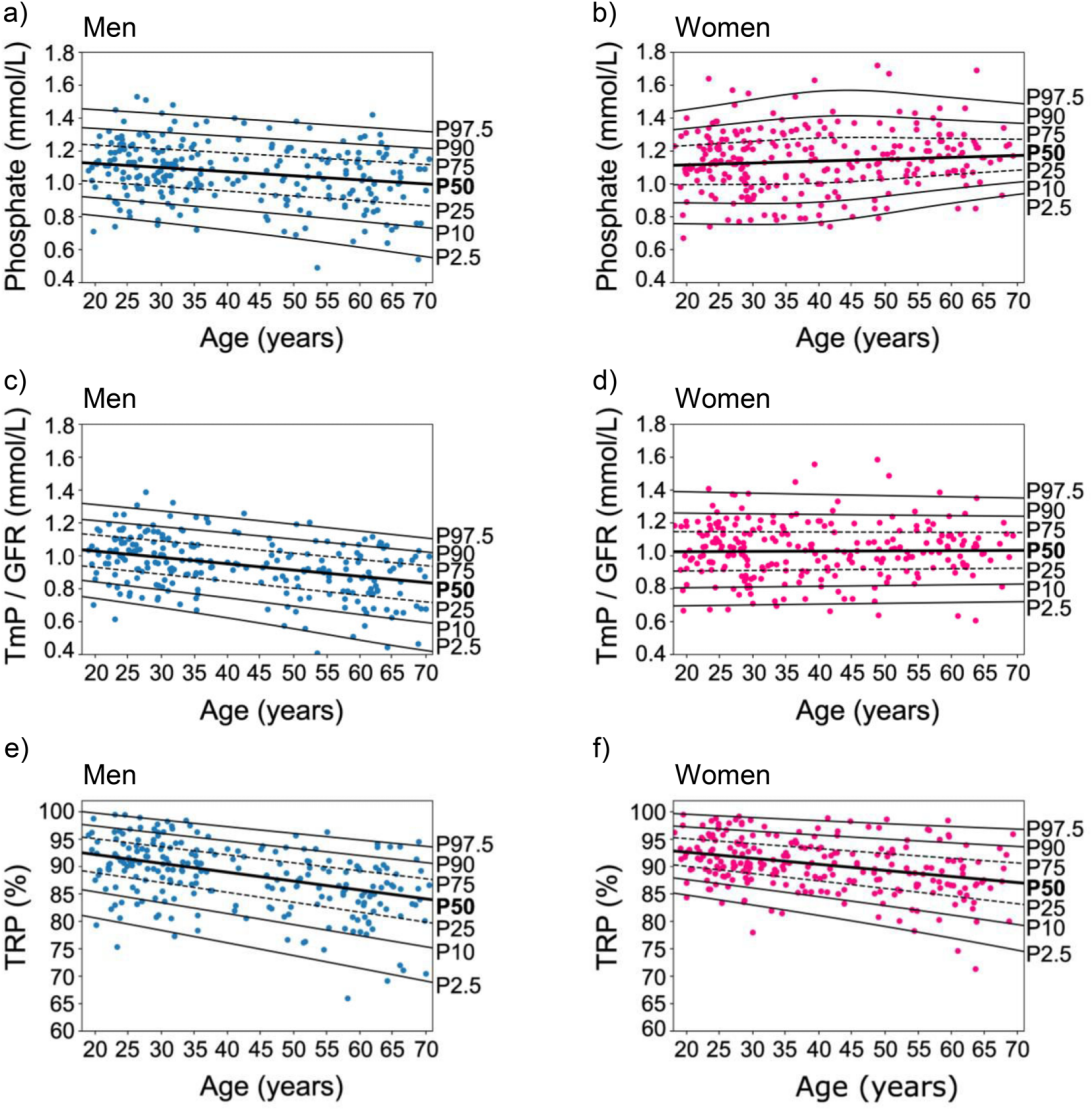
LMS percentiles for serum Pi (A, men; B, women), TmP/GFR (C, men; D, women), and TRP (E, men; F, women) according to age. The 2.5th, 10th, 25th (dotted line), 50th (bold line), 75th (dotted line), 90th, and 97.5th percentiles are given. 1 mmol/l × 3.097 = mg/dl.

The LMS-based percentiles for serum Pi, TmP/GFR, and TRP were higher in women compared to men (each *P* < .001) (Fig. 1 and Supplementary Tables S1–S3). This was mainly attributable to a constant decrease of these parameters in older men, beginning around their sixth decade of life, while no (Pi, TmP/GFR) or only a slight (TRP) decline was observed in women. The median differences between women and men for Pi, TmP/GFR, and TRP were 0.075 mmol/l, 0.093 mmol/l, and 2.2%, respectively (each *P* < .001). It is noteworthy that in women, the variability of serum Pi levels, namely, the range between the 2.5th and 97.5th percentiles, was lower in women aged above 45 years compared to younger women (*P* < .001).

### LMS percentiles for urinary Pi/Crea and reference values for urinary Ca/Crea

**Figure 2:**
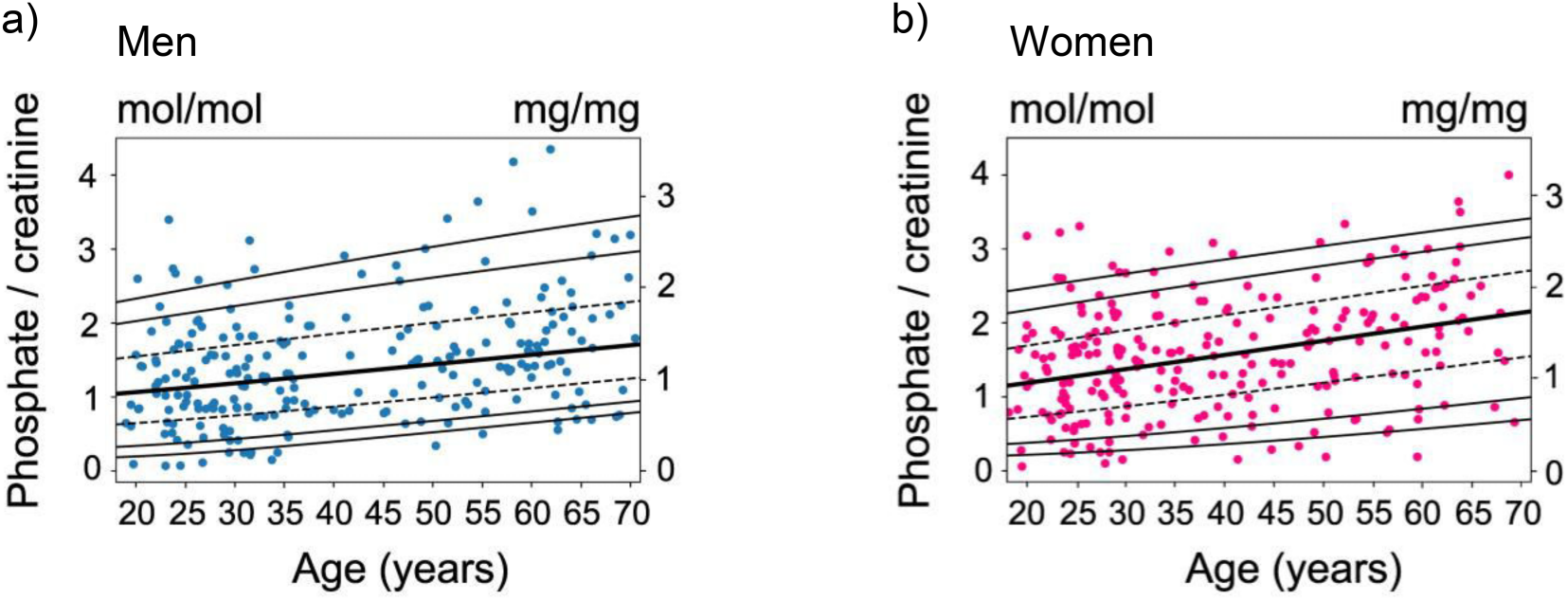
LMS percentiles for urinary Pi/Crea ratios in men (A), and woman (B) according to age. The 2.5th, 10th, 25th (dotted line), 50th (bold line), 75th (dotted line), 90th, and 97.5th percentiles are given. Conversion factor for urinary Pi/Crea 1 mol/mol * 0.84 = 1 mg/mg.

**Figure 3:**
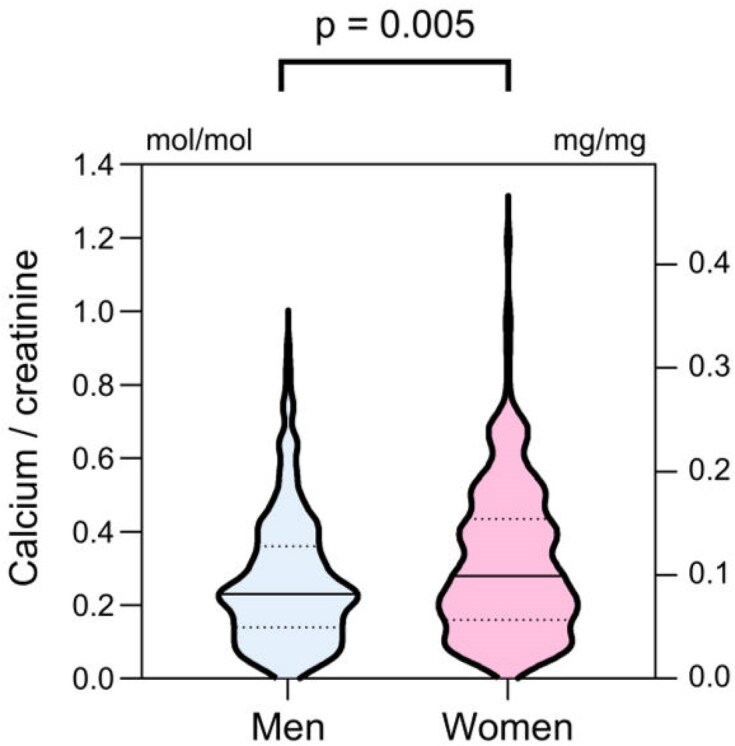
Violin plot showing the distribution of urinary Ca/Crea ratio in men and woman in the HARA cohort. The *P*-value (calculated using Mann–Whitney U test) is indicated. Conversion factor for urinary Ca/Crea 1 mol/mol * 0.35 = 1 mg/mg.

Urine Pi/Crea and Ca/Crea were higher in women than in men (each *P* < .05) (Figs 2 and 3, and Supplementary Tables S4 and S5). Urine Pi/Crea increased with age in both sexes (each *P* < .001), while urine Ca/Crea was independent of age.

### LMS percentiles for plasma iFGF23 and serum sKlotho, and reference values for total FGF23

**Figure 4:**
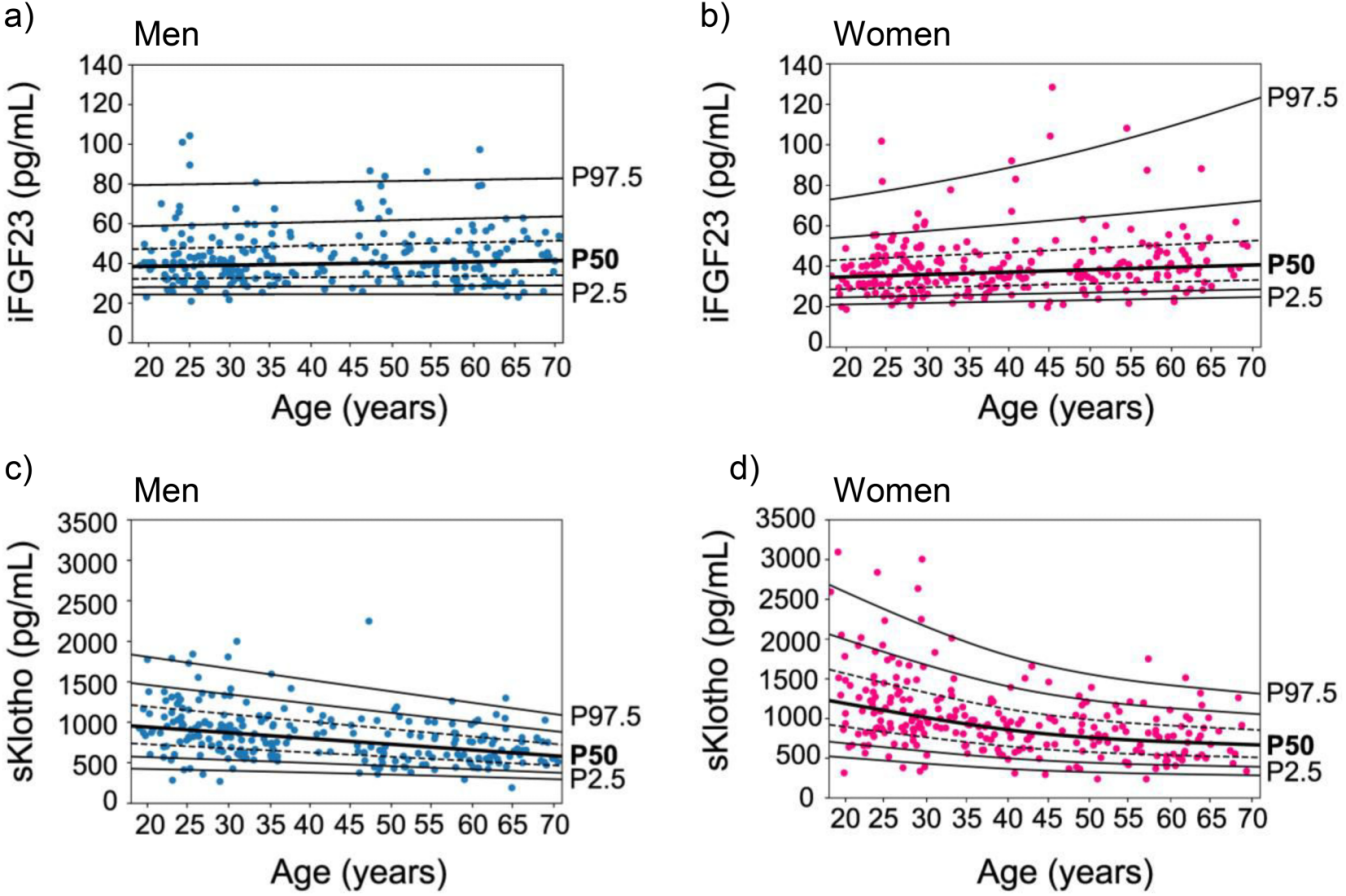
LMS percentiles for plasma iFGF23 (A, men; B, women) and serum sKlotho (C, men; D, women) according to age. The 2.5th, 10th, 25th (dotted line), 50th (bold line), 75th (dotted line), 90th, and 97.5th percentiles are given.

**Figure 5:**
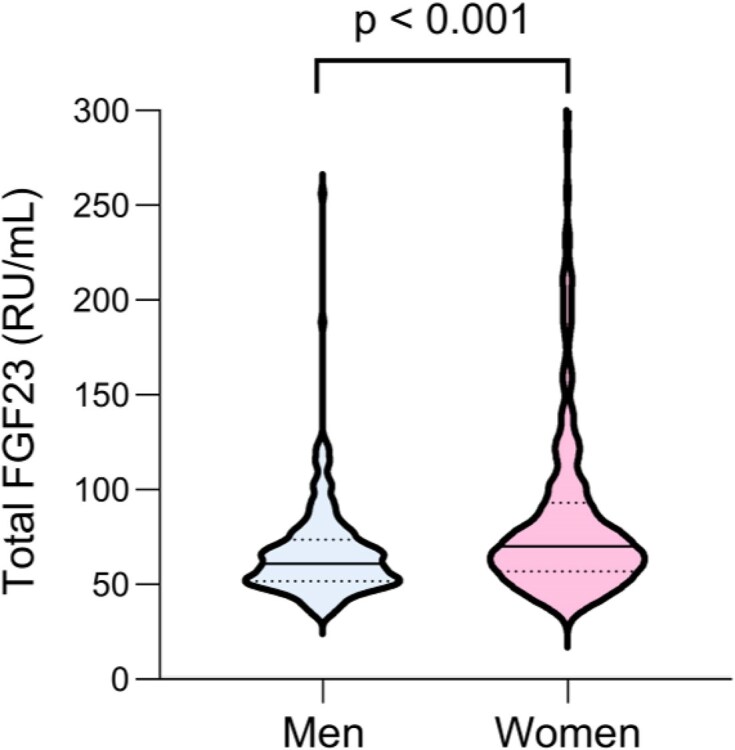
Violin plot showing the distribution of total FGF23 in men and women in the HARA cohort. The *P*-value (calculated using Mann–Whitney U test) is indicated.

**Figure 6:**
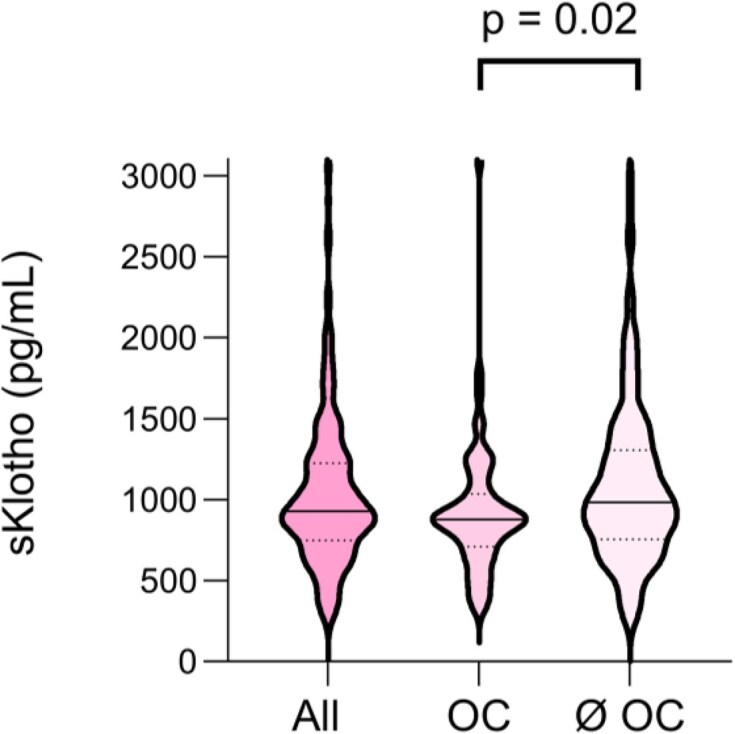
Violin plot showing the distribution of sKlotho in all women aged 18–54 years (left), women aged 18–54 years taking OC (middle), and women aged 18–54 years who do not take OC (right). The *P*-value (calculated using Mann–Whitney U test) is indicated.

The iFGF23 concentrations were higher in men than in women (*P* < .05). iFGF23 concentrations increased with age in women (*P* < .05), which was mainly due to a constant increase in the 97.5th percentile (Fig. 4 and Supplementary Table S6). Total FGF23 concentrations were higher in women than in men (*P* < .001) and independent from age (Fig. 5 and Supplementary Table S7). The percentiles for serum sKlotho were higher in women than in men and decreased with age regardless of sex (Fig. 4 and Supplementary Table S8). Total 62 out of 205 women (30%) aged 18–54 years were on OC. They showed significantly lower median sKlotho concentrations compared to their peers [877 pg/ml (IQR 710; 1035) vs 994 pg/ml (IQR 783; 1312); *P* < .05] (Fig. 6).

### LMS percentiles for plasma iFGF23 to Pi and iFGF23 to sKlotho ratios

**Figure 7:**
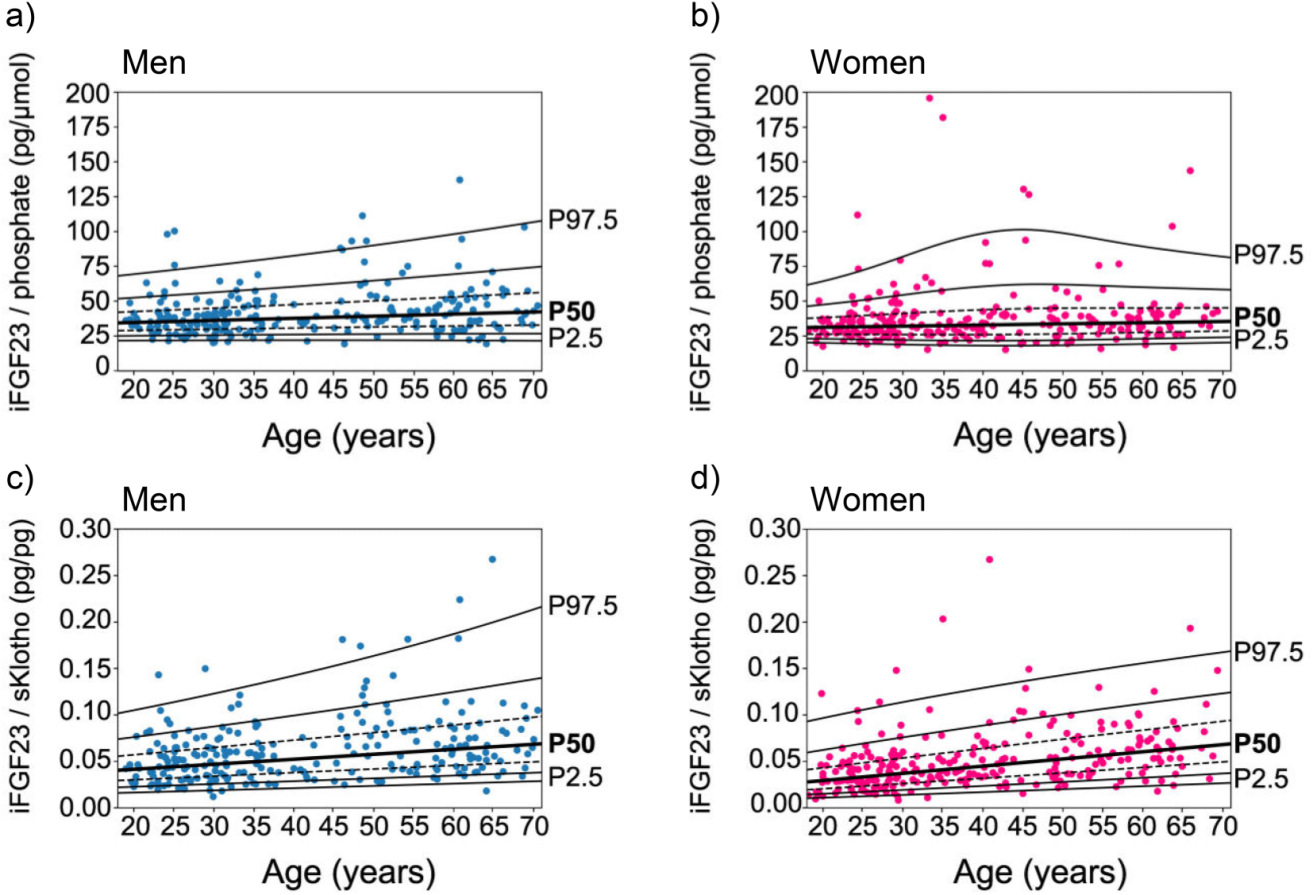
LMS percentiles for iFGF23 to Pi ratio (A, men; B, women) and iFGF23 to sKlotho ratio (C, men; D, women) according to age. The 2.5th, 10th, 25th (dotted line), 50th (bold line), 75th (dotted line), 90th, and 97.5th percentiles are given.

The percentiles for iFGF23/Pi and iFGF23/sKlotho were higher in men compared to women (each *P* < .001). iFGF23/Pi increased with age in men (*P* < .05), and iFGF23/sKlotho increased in age in both sexes (each *P* < .001), which was mainly attributable to an increase in the 90th and 97.5th percentiles and more pronounced in men than in women (Fig. 7 and Supplementary Tables S9 and S10).

### Correlation between parameters of Pi homeostasis

**Figure 8:**
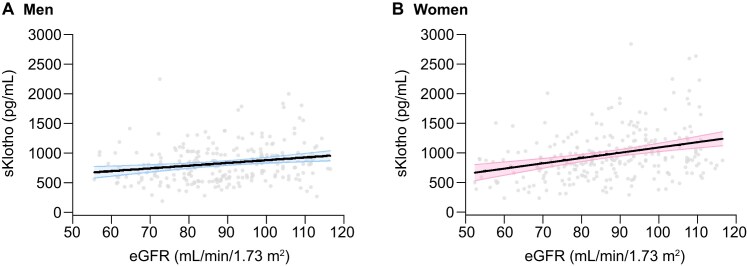
Serum sKlotho as a function of eGFR in men (A, *r* = 0.211, *P* < .001) and women (B, *r* = 0.312, *P* < .001). The mean and 95% confidence intervals are given.

In the whole cohort, in addition to age, serum Pi was also associated with Pi/Crea in urine (cumulative *r*^2^ = 0.19), TRP with eGFR (cumulative *r*^2^ = 0.177), Pi/Crea in urine with serum Pi (cumulative *r*^2^ = 0.26), and iFGF23 with total FGF23 (cumulative *r*^2^ = 0.029) (Table 2), while total FGF23 was only associated with iFGF23 (*r*^2^ = 0.018). In the univariate regression analysis, sKlotho was associated with eGFR regardless of sex (Fig. 8). However, only age remained in the final regression model (*r*^2^ = 0.149). OC use in women was an independent correlate of sKlotho in addition to age in women aged 18–54 years (cumulative *r*^2^ = 0.197). In contrast, presence of A2 albuminuria was not associated with parameters of Pi homeostasis.

**Table 2:** Multivariate linear regression models of variables associated with parameters of Pi homeostasis.

	Serum Pi (mmol/l)		TmP/GFR (mmol/l)		TRP (%)		Pi/Crea in urine (mol/mol)		iFGF23(pg/ml)		Total FGF23 (RU/ml)		sKlotho (pg/ml)		sKlotho women(pg/ml)	
Variable	β(SE)	*P*-value	β(SE)	*P*-value	β(SE)	*P*-value	β(SE)	*P*-value	β(SE)	*P*-value	β(SE)	*P*-value	β(SE)	*P*-value	β(SE)	*P*-value
Age, years	−0.204	<.001	−0.204	<.001	−0.333	<.001	0.34	<.001	0.108	.020	–	–	−0.387	<.001	−0.422	<.001
eGFR, ml/min per 1.73m^2^	–	–	–	–	0.128	.014	–	–	–	–	–	–	–	–	–	–
Pi, mmol/l	–	–	–	–	–	–	0.408	<.001	–	–	–	–	–	–	–	–
U_Pi/Crea_, mol/mol	0.449	<.001	–	–	–	–	–	–	–	–	–	–	–	–	–	–
iFGF23, pg/ml	–	–	–	–	–	–	–	–	–	–	0.133	.004	–	–	–	–
Total FGF23, RU/ml	–	–	–	–	–	–	–	–	0.138	.003	–	–	–	–	–	–
OC	–	–	–	–	–	–	–	–	–	–	–	–	–	–	−0.262	<.001
Cumulative *r*^2^		0.19		0.042		0.177		0.26		0.029		0.018		0.149		0.197

Values represent regression coefficients (β) and significance levels (*P*). Women on OC were aged between 18 and 54 years. TmP, tubular maximum reabsorption of phosphate; U_Pi/Crea_, urinary phosphate-to-creatinine ratio.

## DISCUSSION

To our knowledge, this is the first study to define LMS-based continuous sex-specific, and where appropriate, age-specific reference values for key parameters of Pi homeostasis—iFGF23 and total FGF23 and sKlotho in adults. All parameters examined were sex dependent. In addition, all parameters except total FGF23 and urine Ca/Crea were significantly associated with age. The LMS percentiles shown reflect the distinct physiological sex differences in the laboratory values of these parameters and their profound changes due to aging, which are particularly evident in men in their fifties.

**Figure 9:**
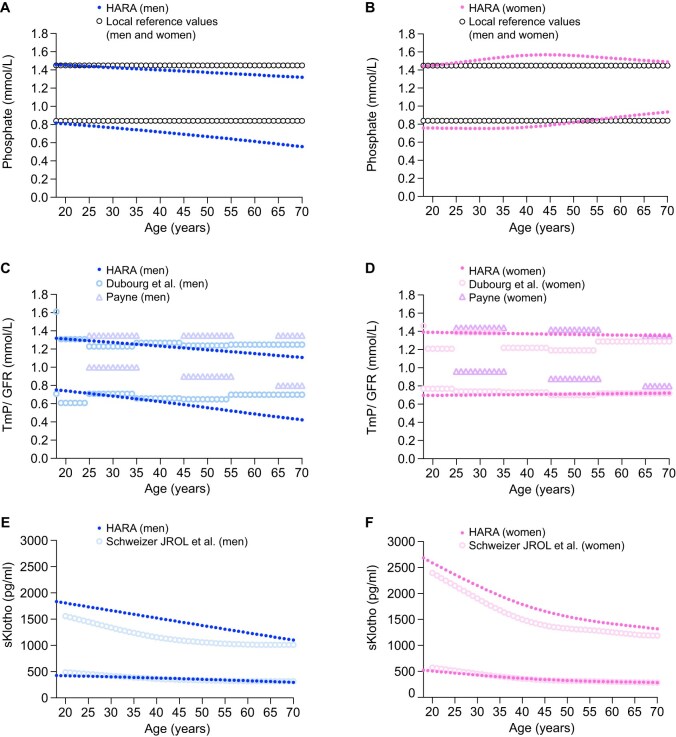
LMS based continuous reference percentiles (2.5th and 97.5th percentiles) in the HARA cohort for serum Pi (A and B), TmP/GFR (C and D), and serum sKlotho (E and F) compared with previously reported reference intervals (upper limit of normal and LLN). The local reference values used at Hannover Medical School [23] for serum Pi (A and B), as well as categorized reference values for TmP/GFR from Derain-Dubourg [29] *et al*. and Payne [30] (C and D) and reference values for sKlotho from Schweizer *et al*. [9] (E and F) are given.

In clinical practice usually uniform reference values for serum Pi are used in women and men irrespectively of age, e.g. the central laboratory of Hannover Medical School specifies a reference range of 0.84–1.45 mmol/l that is largely comparable to the standard values used in the USA [20–23]. We observed pronounced sex-specific, age-dependent differences, which were particularly evident in a strong parallel decline in the lower reference percentiles for serum Pi, TmP/GFR, and TRP, the major determinants of Pi homeostasis, with increasing age in older men but not in women. Figure 9 illustrates the discrepancy between the age- and sex-specific reference values obtained in the present study and our local reference values. Our data indicate that a substantial proportion of males aged more than 50 years (17.4%) would be misdiagnosed to have hypophosphatemia using the same reference values in this age class as done in young adults.

Our findings are in line with four other studies reporting significantly higher serum Pi values in women compared to men, especially above the age of 45 years [6–8, 24]. In the study by Boer *et al*. [6], the serum Pi value in women was 0.16 mg/dl (0.053 mmol/l) higher than in men. This difference was slightly smaller than in the present study (0.075 mmol/l), which may be due to differences in diet between this US and our European cohort and/or ethnicity, as our cohort consisted mainly of Caucasians.

An age-dependent decline in serum Pi, especially above the age of 55 years, was previously reported by Bosman *et al*. [8] and Cirillo *et al*. [24], which was in parallel to the decline in eGFR. However, in the present analysis, eGFR, although being significantly associated with serum Pi in the univariate regression analysis, was excluded from the final regression model, with age and urine Pi/Crea remaining as the only independent correlates of serum Pi. The strong age-related decline in serum Pi, TmP/GFR, and TRP observed in men, accompanied by a parallel increase in urine Pi/Crea, suggests that the decline in serum Pi is most likely due to a decrease in renal Pi reabsorption with increasing age. The latter may be due to the aging process and/or to the age-related decline of growth hormone (GH) levels, as GH stimulates Pi reabsorption in the proximal tubules via upregulation of NPT2a and NPT2c [25–27]. However, since women, unlike men, did not show a significant age-related decrease in Pi and TmP/GFR regardless of eGFR, our findings likely reflect the accelerated aging processes in men compared to women [28].

Our reference values for TmP/GFR were comparable to that of Dubourg *et al*. [29] (Fig. 9). However, in contrast to their study we observed a clear sex and age dependence of TmP/GFR in the HARA cohort. This discrepancy may be at least partly related to the small number of male subjects after the age of 55 in the Dubourg cohort (*n* = 25) [29]. Our TmP/GFR reference values are lower than those reported by Payne [30] (Fig. 9), which may be due to the fact that the latter were derived using the nomogram of Walton and Bijvoet and/or difference in method for determining serum Pi and creatinine.

The commonly used lower limit of normal (LLN) for TRP in adults is 82%, regardless of age and sex [31]. The LMS-based percentiles for TRP in the HARA cohort were age- and sex-dependent. Consequently, a substantial proportion of men (27.9%) and women (10.2%) aged >55 years would be misdiagnosed to have reduced TRP using commonly used LLN. Urine Pi/Crea and urine Ca/Crea have also only been specified as a single reference range for adults to date [32–36]. The LMS-based percentiles for urine Pi/Crea derived from the HARA cohort were clearly age- and sex-dependent and associated with serum Pi, reflecting the physiological relationship between these parameters. Urine Ca/Crea reference values were comparable to that reported in previous studies [34–36], but turned out to be clearly sex-dependent with slightly but significantly higher values in women than men.

In line with a previous study on plasma iFGF23 reference values in adults [37], which used the same ELISA, we found a skewed distribution of iFGF23 values and slightly but significantly higher levels in men than in women (+1.0 pg/ml). Overall iFGF23 reference values were comparable to the abovementioned study but higher or lower compared to two other recent studies, which was probably due to differences in the ELISAs used and/or ethnic differences, since participants of the study of Kato *et al*. were all of Asian descent [38, 39]. In contrast to previous studies evaluating age-categorized iFGF23 values, LMS-based iFGF23 percentiles clearly increased with age in women which was mainly due to a constant rise in the 97.5 percentile [37–39]. It is tempting to speculate that the higher iFGF23 values in older women are driven by their higher serum Pi values. However, none of the parameters of Pi homeostasis examined except of total FGF23 turned out as a significant correlate of iFGF23 in the present study.

A recent study using the same iFGF23 ELISA reported a 100% sensitivity and specificity in differentiating between FGF23-mediated and FGF23-independent hypophosphatemia using a cut-off level of 27 pg/ml [3]. This value corresponds to the 7th and 14th percentile in men and women in the HARA cohort indicating that iFGF23 above these percentiles is inappropriate in the setting of hypophosphatemia, namely indicating FGF23-mediated renal Pi wasting. We also provide LMS-based percentiles for iFGF23 to serum Pi ratio which may be used to evaluate if iFGF23 levels are adequate in relation to actual serum Pi levels in adults as has previously been done in children with nephropathic cystinosis [40]. In contrast to a previous study, we noted clear sex differences in total FGF23 concentrations with higher levels in women than in men which may be due to the fact that the former study evaluated age categories rather than LMS-based reference percentiles [37].

The percentiles for sKlotho in serum were generally higher in women than in men and decreased with age which is in line with previous reports [9, 41–43]. The 2.5th percentile curves for males and females from our cohort and the study of Schweizer *et al*. cohort, both using the same ELISA, align almost perfectly, while the 97.5th percentile curve of the HARA cohort appears to be slightly higher (Fig. 9). In the HARA cohort, women on OC showed considerably lower sKlotho levels than their peers suggesting that estrogen intake impairs renal Klotho synthesis in women of premenopausal age. The age-dependent decline in sKlotho was probably not primarily caused by decreased kidney function as age remained as the only significant correlate of sKlotho in the final regression model. Finally, the presented LMS values for iFGF23 to serum sKlotho may help to evaluate if iFGF23 levels are adequate in relation to reduced sKlotho levels, e.g. in patients with CKD [44].

Our study also has limitations. The vast majority of the study population was of Caucasian descent, thus the results should not be applied to different ethnicities without further research. In addition, sampling was not conducted strictly in the fasting state leading to possible bias, especially with respect to serum Pi values. Nonetheless, differences between fasting and nonfasting serum Pi levels were shown to be rather small, and this better fits to clinical practice where blood samples are often not drawn in the fasting state. Indeed, nonfasting samples were used in recent reference studies in children and guidelines [2, 45, 46]. Variation in dietary Pi intake could induce changes in urinary Pi excretion and Pi-related measures such as FGF23 and 1,25OHD. Thus, it was shown that a Pi-rich diet leads to higher iFGF23 and total FGF23 concentrations in healthy subjects [47]. Pi absorption is further higher from meat-based than from plant-based protein sources. However, in the general population, iFGF23 concentrations showed no differences between urban nonvegetarians, urban vegetarians, and rural vegetarians irrespective of age, sex, presence of diabetes, and BMI [48]. In addition, some studies observed that both iFGF23 and total FGF23 have diurnal variation in humans [47], while others have shown that only iFGF23 displays a diurnal variation [37]. One study determined a 24-h profile of iFGF23 showing an estimated acrophase at 8:30 a.m. and a nadir at 8:30 p.m. with interindividual variations [49]. One limitation of our study is that sampling was performed over a wide time window between 8 a.m. and 2 p.m. irrespective of fasting state, thus we cannot exclude differences in the measures due to circadian rhythm or dietary Pi intake.

Finally, it has been demonstrated that both parameters of iron homeostasis and cardiac function influence FGF23 concentrations [50, 51]. However, these were not examined in the present study. We did, however, exclude patients with anemia or a history of impaired cardiac function in the study.

In conclusion, our data indicate that most key parameters and regulators of Pi homeostasis in adults are age- and sex-dependent. The presented age- and sex-specific LMS values derived from the HARA cohort will enable physicians to calculate standardized patient *z*-scores for these parameters thereby improving the assessment and monitoring of women and men in clinical practice and studies.

## Supplementary Material

sfag147_Supplemental_File

## Data Availability

Some or all datasets generated during and/or analysed during the current study are not publicly available but are available from the corresponding author on reasonable request.
