# Supplementary material for: Age- and sex-specific reference values for phosphate homeostasis parameters—fibroblast growth factor 23 and soluble Klotho
Source: Clin Kidney J. 2026 May 8;19(6):sfag147. doi: 10.1093/ckj/sfag147 (PMC13284703; doi:10.1093/ckj/sfag147)
Supplement: sfag147_Supplemental_File [file sfag147_supplemental_file.pdf]

SUPPLEMENTARY INFORMATION

Age and sex-specific reference values for phosphate homeostasis parameters, fibroblast growth factor 23 and soluble Klotho

Katharina Schermuly[1], Hannah Weber[1], Anna Tschirner[1], Thomas Rebe[2], Dieter Haffner[1], Maren Leifheit-Nestler[1]

[1]Department of Pediatric Kidney, Liver, Metabolic and Neurological Diseases, Hannover Medical School, Carl-Neuberg-Str. 1, 30625 Hannover, Germany

[2]Department of Occupational Medicine, Hannover Medical School, Carl-Neuberg-Str.1, 30625 Hannover, Germany

Correspondence: Prof. Dr. Dieter Haffner, MD, Department of Pediatric Kidney, Liver, Metabolic and Neurological Diseases, Hannover Medical School, Carl-Neuberg-Str. 1, D-30625 Hannover, Germany. E-Mail: haffner.dieter@mh-hannover.de.

Supplementary Table S1. Age-specific percentile limits and LMS values for serum phosphate (mmol/L) in men and women

Age (yrs)	Men					Women				
	2.5th	*50th (M)*	*97.5th*	*L*	*S*	*2.5th*	*50th (M)*	*97.5th*	*L*	*S*
18	0.82	1.13	1.46	0.8446	0.1450	0.76	1.11	1.44	1.2576	0.1554
19	0.81	1.13	1.45	0.8639	0.1456	0.76	1.11	1.45	1.2283	0.1567
20	0.81	1.12	1.45	0.8833	0.1463	0.76	1.12	1.45	1.1991	0.1581
21	0.80	1.12	1.45	0.9027	0.1470	0.76	1.12	1.46	1.1698	0.1594
22	0.80	1.12	1.45	0.9220	0.1477	0.76	1.12	1.46	1.1405	0.1608
23	0.80	1.12	1.44	0.9414	0.1484	0.76	1.12	1.47	1.1113	0.1622
24	0.80	1.11	1.44	0.9608	0.1491	0.76	1.12	1.48	1.0820	0.1636
25	0.79	1.11	1.44	0.9801	0.1498	0.76	1.12	1.48	1.0527	0.1650
26	0.78	1.11	1.44	0.9995	0.1505	0.75	1.12	1.49	1.0234	0.1665
27	0.78	1.11	1.43	1.0189	0.1512	0.75	1.12	1.49	0.9942	0.1680
28	0.77	1.10	1.43	1.0382	0.1519	0.75	1.12	1.50	0.9649	0.1694
29	0.77	1.10	1.43	1.0576	0.1526	0.75	1.13	1.51	0.9356	0.1709
30	0.76	1.10	1.43	1.0770	0.1533	0.75	1.13	1.51	0.9064	0.1723
31	0.76	1.10	1.42	1.0963	0.1540	0.75	1.13	1.52	0.8771	0.1736
32	0.75	1.09	1.42	1.1157	0.1548	0.75	1.13	1.53	0.8478	0.1748
33	0.75	1.09	1.42	1.1350	0.1555	0.75	1.13	1.53	0.8186	0.1760
34	0.75	1.09	1.42	1.1544	0.1562	0.75	1.13	1.54	0.7893	0.1770
35	0.74	1.09	1.41	1.1738	0.1569	0.76	1.13	1.54	0.7600	0.1778
36	0.74	1.08	1.41	1.1931	0.1577	0.76	1.13	1.55	0.7307	0.1785
37	0.73	1.08	1.41	1.2125	0.1584	0.76	1.14	1.55	0.7015	0.1789
38	0.73	1.08	1.41	1.2319	0.1592	0.76	1.14	1.56	0.6722	0.1791
39	0.72	1.08	1.40	1.2512	0.1599	0.76	1.14	1.56	0.6429	0.1791
40	0.72	1.07	1.40	1.2706	0.1606	0.77	1.14	1.56	0.6137	0.1788
41	0.71	1.07	1.40	1.2900	0.1614	0.77	1.14	1.57	0.5844	0.1783
42	0.71	1.07	1.40	1.3093	0.1622	0.78	1.14	1.57	0.5551	0.1775
43	0.70	1.07	1.39	1.3287	0.1629	0.78	1.14	1.57	0.5259	0.1765
44	0.70	1.06	1.39	1.3481	0.1637	0.78	1.14	1.57	0.4966	0.1753
45	0.69	1.06	1.39	1.3674	0.1644	0.79	1.14	1.57	0.4673	0.1738
46	0.69	1.06	1.38	1.3868	0.1652	0.80	1.15	1.57	0.4380	0.1722
47	0.68	1.06	1.38	1.4061	0.1660	0.80	1.15	1.57	0.4088	0.1703
48	0.68	1.05	1.38	1.4255	0.1668	0.81	1.15	1.57	0.3795	0.1683
49	0.67	1.05	1.38	1.4449	0.1675	0.81	1.15	1.56	0.3502	0.1662
50	0.67	1.05	1.37	1.4642	0.1683	0.82	1.15	1.56	0.3210	0.1639
51	0.66	1.05	1.37	1.4836	0.1691	0.83	1.15	1.56	0.2917	0.1615
52	0.66	1.04	1.37	1.5030	0.1699	0.83	1.15	1.55	0.2624	0.1591
53	0.65	1.04	1.37	1.5223	0.1707	0.84	1.15	1.55	0.2332	0.1566
54	0.65	1.04	1.36	1.5417	0.1715	0.85	1.16	1.55	0.2039	0.1541
55	0.64	1.04	1.36	1.5611	0.1723	0.85	1.16	1.54	0.1746	0.1515
56	0.64	1.03	1.36	1.5804	0.1731	0.86	1.16	1.54	0.1453	0.1491
57	0.63	1.03	1.35	1.5998	0.1739	0.86	1.16	1.54	0.1161	0.1466
58	0.63	1.03	1.35	1.6192	0.1747	0.87	1.16	1.53	0.0868	0.1442

59	0.62	1.03	1.35	1.6385	0.1756	0.88	1.16	1.53	0.0575	0.1419
60	0.61	1.02	1.35	1.6579	0.1764	0.88	1.16	1.53	0.0283	0.1396
61	0.61	1.02	1.34	1.6773	0.1772	0.89	1.16	1.52	-0.0010	0.1374
62	0.60	1.02	1.34	1.6966	0.1780	0.89	1.16	1.52	-0.0303	0.1352
63	0.60	1.02	1.34	1.7160	0.1789	0.90	1.16	1.51	-0.0595	0.1330
64	0.59	1.01	1.34	1.7353	0.1797	0.90	1.17	1.51	-0.0888	0.1309
65	0.59	1.01	1.33	1.7547	0.1805	0.91	1.17	1.51	-0.1181	0.1288
66	0.58	1.01	1.33	1.7741	0.1814	0.92	1.17	1.50	-0.1474	0.1267
67	0.57	1.01	1.33	1.7934	0.1822	0.92	1.17	1.50	-0.1766	0.1246
68	0.57	1.00	1.32	1.8128	0.1831	0.93	1.17	1.50	-0.2059	0.1226
69	0.56	1.00	1.32	1.8322	0.1840	0.93	1.17	1.49	-0.2352	0.1206
70	0.56	1.00	1.32	1.8515	0.1848	0.94	1.17	1.49	-0.2644	0.1186

To calculate the age-related z-score for serum phosphate, the determined concentration x together with the corresponding age and sex-related values for L, M, and S are used according to the formula $z = [(x/M)^{L}-1]/S \times L$. L, skewness; M, median; S, coefficient of variation.

Supplementary Table S2. Age-specific percentile limits and LMS values for TmP/GFR (mmol/L) in men and women

Age (yrs)	Men					Women				
	2.5th	*50th (M)*	*97.5th*	*L*	*S*	*2.5th*	*50th (M)*	*97.5th*	*L*	*S*
18	0.75	1.04	1.32	1.0153	0.1394	0.70	1.03	1.39	0.6915	0.1734
19	0.75	1.03	1.32	1.0363	0.1403	0.70	1.03	1.39	0.6960	0.1731
20	0.74	1.03	1.31	1.0573	0.1412	0.70	1.03	1.39	0.7005	0.1727
21	0.74	1.03	1.31	1.0783	0.1422	0.70	1.03	1.39	0.7050	0.1724
22	0.73	1.02	1.30	1.0993	0.1431	0.70	1.03	1.39	0.7095	0.1720
23	0.72	1.02	1.30	1.1202	0.1440	0.70	1.03	1.39	0.7140	0.1717
24	0.72	1.01	1.30	1.1412	0.1450	0.70	1.03	1.39	0.7186	0.1713
25	0.71	1.01	1.29	1.1622	0.1459	0.70	1.03	1.39	0.7231	0.1710
26	0.71	1.01	1.29	1.1832	0.1469	0.70	1.03	1.39	0.7276	0.1706
27	0.70	1.00	1.29	1.2042	0.1478	0.70	1.03	1.38	0.7321	0.1703
28	0.70	1.00	1.28	1.2252	0.1488	0.70	1.03	1.38	0.7366	0.1700
29	0.69	0.99	1.28	1.2462	0.1498	0.70	1.03	1.38	0.7411	0.1696
30	0.68	0.99	1.27	1.2672	0.1507	0.70	1.03	1.38	0.7456	0.1693
31	0.68	0.99	1.27	1.2882	0.1517	0.70	1.03	1.38	0.7501	0.1689
32	0.67	0.98	1.27	1.3092	0.1527	0.70	1.03	1.38	0.7546	0.1686
33	0.67	0.98	1.26	1.3302	0.1537	0.70	1.03	1.38	0.7591	0.1682
34	0.66	0.98	1.26	1.3511	0.1547	0.70	1.03	1.38	0.7636	0.1679
35	0.65	0.97	1.25	1.3721	0.1557	0.70	1.03	1.38	0.7681	0.1675
36	0.65	0.97	1.25	1.3931	0.1568	0.70	1.03	1.38	0.7726	0.1672
37	0.64	0.96	1.25	1.4141	0.1578	0.71	1.03	1.38	0.7771	0.1669
38	0.64	0.96	1.24	1.4351	0.1588	0.71	1.03	1.38	0.7816	0.1665
39	0.63	0.96	1.24	1.4561	0.1598	0.71	1.03	1.37	0.7861	0.1662
40	0.62	0.95	1.23	1.4771	0.1609	0.71	1.03	1.37	0.7906	0.1659
41	0.62	0.95	1.23	1.4981	0.1619	0.71	1.03	1.37	0.7951	0.1655
42	0.61	0.94	1.23	1.5191	0.1630	0.71	1.03	1.37	0.7996	0.1652
43	0.60	0.94	1.22	1.5401	0.1641	0.71	1.03	1.37	0.8041	0.1648
44	0.60	0.94	1.22	1.5611	0.1651	0.71	1.03	1.37	0.8086	0.1645
45	0.59	0.93	1.21	1.5821	0.1662	0.71	1.03	1.37	0.8131	0.1642
46	0.58	0.93	1.21	1.6031	0.1673	0.71	1.03	1.37	0.8176	0.1638
47	0.58	0.93	1.21	1.6240	0.1684	0.71	1.03	1.37	0.8221	0.1635
48	0.57	0.92	1.20	1.6450	0.1695	0.71	1.03	1.37	0.8266	0.1632
49	0.56	0.92	1.20	1.6660	0.1706	0.71	1.03	1.37	0.8311	0.1628
50	0.56	0.91	1.19	1.6870	0.1717	0.71	1.03	1.37	0.8356	0.1625
51	0.55	0.91	1.19	1.7080	0.1729	0.71	1.03	1.37	0.8401	0.1622
52	0.54	0.91	1.18	1.7290	0.1740	0.71	1.03	1.37	0.8446	0.1619
53	0.54	0.90	1.18	1.7500	0.1751	0.71	1.03	1.36	0.8491	0.1615
54	0.53	0.90	1.18	1.7710	0.1763	0.71	1.03	1.36	0.8537	0.1612
55	0.52	0.89	1.17	1.7920	0.1774	0.71	1.03	1.36	0.8582	0.1609
56	0.52	0.89	1.17	1.8130	0.1786	0.71	1.03	1.36	0.8627	0.1605
57	0.51	0.89	1.16	1.8340	0.1797	0.72	1.03	1.36	0.8672	0.1602
58	0.50	0.88	1.16	1.8550	0.1809	0.72	1.03	1.36	0.8717	0.1599

59	0.49	0.88	1.16	1.8759	0.1821	0.72	1.03	1.36	0.8762	0.1596
60	0.49	0.88	1.15	1.8969	0.1833	0.72	1.03	1.36	0.8807	0.1592
61	0.48	0.87	1.15	1.9179	0.1845	0.72	1.03	1.36	0.8852	0.1589
62	0.47	0.87	1.14	1.9389	0.1857	0.72	1.03	1.36	0.8897	0.1586
63	0.47	0.86	1.14	1.9599	0.1869	0.72	1.03	1.36	0.8942	0.1583
64	0.46	0.86	1.13	1.9809	0.1881	0.72	1.03	1.36	0.8987	0.1579
65	0.46	0.86	1.13	2.0019	0.1894	0.72	1.03	1.36	0.9032	0.1576
66	0.45	0.85	1.13	2.0229	0.1906	0.72	1.03	1.36	0.9077	0.1573
67	0.44	0.85	1.12	2.0439	0.1918	0.72	1.03	1.35	0.9122	0.1570
68	0.44	0.84	1.12	2.0649	0.1931	0.72	1.03	1.35	0.9167	0.1567
69	0.43	0.84	1.11	2.0859	0.1944	0.72	1.03	1.35	0.9212	0.1563
70	0.42	0.84	1.11	2.1069	0.1956	0.72	1.03	1.35	0.9257	0.1560

To calculate the age-related z-score for TmP/GFR, the determined concentration x together with the corresponding age and sex-related values for L, M, and S are used according to the formula $z = [(x/M)^L-1]/S\times L$. L, skewness; M, median; S, coefficient of variation.

Supplementary Table S3. Age-specific percentile limits and LMS values for TRP (%) in men and women

Age (yrs)	Men					Women				
	2.5th	*50th (M)*	*97.5th*	*L*	*S*	*2.5th*	*50th (M)*	*97.5th*	*L*	*S*
18	81	93	100	5.1416	0.0489	85	93	100	2.4943	0.0396
19	81	92	100	5.1209	0.0492	85	93	100	2.5012	0.0400
20	81	92	100	5.1001	0.0495	85	93	100	2.5081	0.0404
21	80	92	100	5.0794	0.0499	85	93	100	2.5150	0.0407
22	80	92	100	5.0587	0.0502	85	92	99	2.5219	0.0411
23	80	92	99	5.0379	0.0505	84	92	99	2.5288	0.0415
24	80	92	99	5.0172	0.0509	84	92	99	2.5357	0.0418
25	80	91	99	4.9965	0.0512	84	92	99	2.5425	0.0422
26	79	91	99	4.9757	0.0516	84	92	99	2.5494	0.0426
27	79	91	99	4.9550	0.0519	84	92	99	2.5563	0.0430
28	79	91	99	4.9343	0.0523	83	92	99	2.5632	0.0434
29	79	91	99	4.9135	0.0526	83	92	99	2.5701	0.0438
30	78	91	99	4.8928	0.0530	83	92	99	2.5770	0.0441
31	78	90	98	4.8721	0.0533	83	91	99	2.5839	0.0445
32	78	90	98	4.8513	0.0537	83	91	99	2.5908	0.0449
33	78	90	98	4.8306	0.0540	82	91	99	2.5977	0.0454
34	78	90	98	4.8099	0.0544	82	91	99	2.6046	0.0458
35	77	90	98	4.7891	0.0548	82	91	99	2.6115	0.0462
36	77	90	98	4.7684	0.0551	82	91	99	2.6184	0.0466
37	77	90	98	4.7477	0.0555	82	91	99	2.6253	0.0470
38	77	89	98	4.7269	0.0559	82	91	99	2.6322	0.0474
39	76	89	97	4.7062	0.0562	81	91	98	2.6391	0.0479
40	76	89	97	4.6855	0.0566	81	90	98	2.6460	0.0483
41	76	89	97	4.6647	0.0570	81	90	98	2.6529	0.0487
42	76	89	97	4.6440	0.0574	81	90	98	2.6598	0.0492
43	75	89	97	4.6233	0.0578	81	90	98	2.6667	0.0496
44	75	88	97	4.6025	0.0581	80	90	98	2.6736	0.0501
45	75	88	97	4.5818	0.0585	80	90	98	2.6805	0.0505
46	75	88	97	4.5611	0.0589	80	90	98	2.6874	0.0510
47	75	88	96	4.5403	0.0593	80	90	98	2.6943	0.0514
48	74	88	96	4.5196	0.0597	80	90	98	2.7012	0.0519
49	74	88	96	4.4989	0.0601	79	89	98	2.7081	0.0524
50	74	87	96	4.4781	0.0605	79	89	98	2.7150	0.0528
51	74	87	96	4.4574	0.0609	79	89	98	2.7219	0.0533
52	73	87	96	4.4367	0.0613	79	89	98	2.7288	0.0538
53	73	87	96	4.4159	0.0617	79	89	98	2.7357	0.0543
54	73	87	96	4.3952	0.0621	78	89	98	2.7426	0.0548
55	73	87	96	4.3745	0.0626	78	89	98	2.7495	0.0553
56	72	86	95	4.3537	0.0630	78	89	98	2.7564	0.0558
57	72	86	95	4.3330	0.0634	78	89	98	2.7633	0.0563
58	72	86	95	4.3123	0.0638	77	88	97	2.7702	0.0568

59	72	86	95	4.2915	0.0643	77	88	97	2.7771	0.0573
60	71	86	95	4.2708	0.0647	77	88	97	2.7840	0.0578
61	71	86	95	4.2500	0.0651	77	88	97	2.7909	0.0583
62	71	85	95	4.2293	0.0655	77	88	97	2.7978	0.0589
63	71	85	95	4.2086	0.0660	76	88	97	2.8047	0.0594
64	71	85	94	4.1878	0.0664	76	88	97	2.8116	0.0599
65	70	85	94	4.1671	0.0669	76	88	97	2.8185	0.0605
66	70	85	94	4.1464	0.0673	76	88	97	2.8254	0.0610
67	70	85	94	4.1256	0.0678	75	87	97	2.8323	0.0616
68	70	84	94	4.1049	0.0682	75	87	97	2.8392	0.0621
69	69	84	94	4.0842	0.0687	75	87	97	2.8461	0.0627
70	69	84	94	4.0634	0.0691	75	87	97	2.8530	0.0632

To calculate the age-related z-score for TRP, the determined concentration x together with the corresponding age-related values for L, M, and S are used according to the formula $z = [(x/M)^L-1]/S \times L$. L, skewness; M, median; S, coefficient of variation.

Supplementary Table S4. Age-specific percentile limits and LMS values for urinary Pi/Crea ratio (mol/mol) in men and women

Age (yrs)	Men					Women				
	5th	*50th (M)*	*95th*	*L*	*S*	*5th*	*50th (M)*	*95th*	*L*	*S*
18	0.19	1.03	2.28	0.7257	0.6518	0.21	1.13	2.43	0.8146	0.6448
19	0.19	1.04	2.31	0.7142	0.6471	0.21	1.14	2.45	0.8322	0.6426
20	0.20	1.06	2.33	0.7027	0.6425	0.22	1.16	2.47	0.8499	0.6404
21	0.20	1.07	2.36	0.6913	0.6379	0.22	1.17	2.49	0.8675	0.6382
22	0.21	1.08	2.38	0.6798	0.6333	0.23	1.19	2.51	0.8851	0.6360
23	0.22	1.09	2.40	0.6683	0.6287	0.23	1.21	2.53	0.9028	0.6338
24	0.23	1.11	2.43	0.6569	0.6242	0.24	1.22	2.55	0.9204	0.6316
25	0.23	1.12	2.45	0.6454	0.6197	0.25	1.24	2.57	0.9381	0.6295
26	0.24	1.13	2.48	0.6339	0.6152	0.25	1.25	2.59	0.9557	0.6273
27	0.25	1.15	2.50	0.6225	0.6108	0.26	1.27	2.61	0.9733	0.6252
28	0.26	1.16	2.53	0.6110	0.6064	0.27	1.29	2.63	0.9910	0.6231
29	0.27	1.17	2.55	0.5995	0.6020	0.27	1.30	2.65	1.0086	0.6209
30	0.28	1.18	2.57	0.5880	0.5977	0.28	1.32	2.67	1.0263	0.6188
31	0.29	1.20	2.60	0.5766	0.5934	0.29	1.33	2.69	1.0439	0.6167
32	0.30	1.21	2.62	0.5651	0.5891	0.29	1.35	2.71	1.0615	0.6146
33	0.31	1.22	2.64	0.5536	0.5849	0.30	1.36	2.72	1.0792	0.6125
34	0.32	1.24	2.67	0.5422	0.5807	0.31	1.38	2.74	1.0968	0.6104
35	0.34	1.25	2.70	0.5307	0.5765	0.32	1.40	2.76	1.1145	0.6083
36	0.35	1.26	2.72	0.5192	0.5724	0.33	1.41	2.78	1.1321	0.6062
37	0.36	1.27	2.74	0.5078	0.5682	0.33	1.43	2.80	1.1497	0.6042
38	0.37	1.29	2.76	0.4963	0.5641	0.34	1.44	2.82	1.1674	0.6021
39	0.38	1.30	2.78	0.4848	0.5601	0.35	1.46	2.84	1.1850	0.6000
40	0.40	1.31	2.81	0.4734	0.5561	0.36	1.48	2.86	1.2027	0.5980
41	0.41	1.33	2.83	0.4619	0.5521	0.37	1.49	2.88	1.2203	0.5959
42	0.42	1.34	2.85	0.4504	0.5481	0.38	1.51	2.89	1.2379	0.5939
43	0.43	1.35	2.88	0.4390	0.5441	0.39	1.52	2.91	1.2556	0.5919
44	0.45	1.36	2.90	0.4275	0.5402	0.40	1.54	2.93	1.2732	0.5898
45	0.46	1.38	2.92	0.4160	0.5363	0.41	1.56	2.95	1.2909	0.5878
46	0.47	1.39	2.94	0.4046	0.5325	0.42	1.57	2.97	1.3085	0.5858
47	0.48	1.40	2.96	0.3931	0.5286	0.43	1.59	2.99	1.3261	0.5838
48	0.50	1.42	2.99	0.3816	0.5248	0.44	1.60	3.01	1.3438	0.5818
49	0.51	1.43	3.01	0.3702	0.5211	0.45	1.62	3.02	1.3614	0.5798
50	0.52	1.44	3.03	0.3587	0.5173	0.46	1.64	3.04	1.3791	0.5779
51	0.54	1.46	3.05	0.3472	0.5136	0.47	1.65	3.06	1.3967	0.5759
52	0.55	1.47	3.07	0.3357	0.5099	0.48	1.67	3.08	1.4143	0.5739
53	0.56	1.48	3.09	0.3243	0.5062	0.49	1.68	3.10	1.4320	0.5720
54	0.57	1.49	3.12	0.3128	0.5026	0.50	1.70	3.12	1.4496	0.5700
55	0.59	1.51	3.14	0.3013	0.4990	0.51	1.72	3.13	1.4673	0.5681
56	0.60	1.52	3.16	0.2899	0.4954	0.52	1.73	3.15	1.4849	0.5661
57	0.61	1.53	3.18	0.2784	0.4918	0.53	1.75	3.17	1.5025	0.5642

58	0.63	1.55	3.20	0.2669	0.4883	0.54	1.76	3.19	1.5202	0.5622
59	0.64	1.56	3.22	0.2555	0.4847	0.56	1.78	3.20	1.5378	0.5603
60	0.65	1.57	3.24	0.2440	0.4813	0.57	1.80	3.22	1.5555	0.5584
61	0.67	1.58	3.26	0.2325	0.4778	0.58	1.81	3.24	1.5731	0.5565
62	0.68	1.60	3.28	0.2211	0.4744	0.59	1.83	3.26	1.5907	0.5546
63	0.69	1.61	3.30	0.2096	0.4709	0.60	1.84	3.28	1.6084	0.5527
64	0.70	1.62	3.32	0.1981	0.4675	0.61	1.86	3.29	1.6260	0.5508
65	0.72	1.64	3.34	0.1867	0.4642	0.63	1.88	3.31	1.6437	0.5489
66	0.73	1.65	3.36	0.1752	0.4608	0.64	1.89	3.33	1.6613	0.5471
67	0.74	1.66	3.38	0.1637	0.4575	0.65	1.91	3.35	1.6789	0.5452
68	0.76	1.67	3.40	0.1523	0.4542	0.66	1.92	3.36	1.6966	0.5433
69	0.77	1.69	3.42	0.1408	0.4510	0.67	1.94	3.380	1.7142	0.5415
70	0.78	1.70	3.44	0.1293	0.4477	0.69	1.96	3.40	1.7319	0.5396

To calculate the age-related z-score for urinary Pi/Crea ratio, the determined concentration x together with the corresponding age-related values for L, M, and S are used according to the formula $z = [(x/M)^L - 1]/S \times L$. L, skewness; M, median; S, coefficient of variation.

Supplementary Table S5. Age-specific percentile limits and LMS values for urinary Ca/Crea (mol/mol) in men and women

Age (yrs)	Men					
	5th	*50th*	*95th*	*L*	*M*	*S*
18-70	0.05	0.22	0.63	-52.0060	10.2656	0.0217
	Women					
	5th	*50th*	*95th*	*L*	*M*	*S*
18-70	0.07	0.27	0.77	-44.0795	10.3313	0.0271

Before computing the age-adjusted z-score for urinary Ca/Crea ratio, 10 must be added to the subject´s measured concentration x due to mathematical requirements of the RefCurv software. The formula that should be used is $z = [((x+10)/M)^{L}-1]/SxL$. L, skewness; M, median; S, coefficient of variation.

Supplementary Table S6. Age-specific percentile limits and LMS values for plasma intact FGF23 (pg/mL) in men and women

Age (yrs)	Men					Women				
	2.5th	*50th (M)*	*97.5th*	*L*	*S*	*2.5th*	*50th (M)*	*97.5th*	*L*	*S*
18	24.38	38.41	79.41	-0.8082	0.2803	20.99	34.49	72.94	-0.6696	0.3005
19	24.39	38.47	79.47	-0.8010	0.2807	21.06	34.61	73.54	-0.6774	0.3012
20	24.39	38.53	79.53	-0.7939	0.2812	21.13	34.73	74.15	-0.6853	0.3018
21	24.40	38.59	79.60	-0.7868	0.2816	21.21	34.85	74.77	-0.6931	0.3025
22	24.40	38.65	79.66	-0.7797	0.2821	21.28	34.96	75.40	-0.7009	0.3031
23	24.41	38.71	79.73	-0.7726	0.2825	21.35	35.08	76.03	-0.7088	0.3038
24	24.41	38.76	79.79	-0.7655	0.2830	21.42	35.20	76.68	-0.7166	0.3045
25	24.42	38.82	79.85	-0.7584	0.2834	21.49	35.32	77.34	-0.7245	0.3051
26	24.42	38.88	79.92	-0.7513	0.2839	21.56	35.44	78.01	-0.7323	0.3058
27	24.43	38.94	79.98	-0.7442	0.2843	21.63	35.56	78.69	-0.7401	0.3065
28	24.43	39.00	80.05	-0.7371	0.2848	21.70	35.68	79.39	-0.7480	0.3071
29	24.44	39.06	80.11	-0.7300	0.2853	21.77	35.79	80.09	-0.7558	0.3078
30	24.44	39.11	80.17	-0.7228	0.2857	21.85	35.91	80.81	-0.7637	0.3085
31	24.45	39.17	80.24	-0.7157	0.2862	21.92	36.03	81.54	-0.7715	0.3092
32	24.45	39.23	80.30	-0.7086	0.2866	21.99	36.15	82.28	-0.7793	0.3098
33	24.45	39.29	80.37	-0.7015	0.2871	22.06	36.27	83.03	-0.7872	0.3105
34	24.45	39.35	80.43	-0.6944	0.2875	22.13	36.39	83.80	-0.7950	0.3112
35	24.46	39.41	80.50	-0.6873	0.2880	22.20	36.51	84.58	-0.8029	0.3119
36	24.46	39.46	80.56	-0.6802	0.2885	22.27	36.63	85.37	-0.8107	0.3126
37	24.46	39.52	80.63	-0.6731	0.2889	22.34	36.74	86.18	-0.8185	0.3132
38	24.46	39.58	80.69	-0.6660	0.2894	22.42	36.86	87.01	-0.8264	0.3139
39	24.47	39.64	80.76	-0.6589	0.2898	22.49	36.98	87.85	-0.8342	0.3146
40	24.47	39.70	80.82	-0.6517	0.2903	22.56	37.10	88.70	-0.8421	0.3153
41	24.47	39.75	80.89	-0.6446	0.2908	22.63	37.22	89.57	-0.8499	0.3160
42	24.47	39.81	80.95	-0.6375	0.2912	22.70	37.34	90.45	-0.8578	0.3167
43	24.47	39.87	81.02	-0.6304	0.2917	22.77	37.46	91.36	-0.8656	0.3174
44	24.47	39.93	81.08	-0.6233	0.2922	22.84	37.57	92.27	-0.8734	0.3181
45	24.47	39.99	81.15	-0.6162	0.2926	22.91	37.69	93.21	-0.8813	0.3188
46	24.47	40.05	81.21	-0.6091	0.2931	22.99	37.81	94.16	-0.8891	0.3195
47	24.47	40.10	81.28	-0.6020	0.2936	23.06	37.93	95.13	-0.8970	0.3202
48	24.47	40.16	81.34	-0.5949	0.2940	23.13	38.05	96.11	-0.9048	0.3209
49	24.47	40.22	81.41	-0.5878	0.2945	23.20	38.17	97.12	-0.9126	0.3216
50	24.47	40.28	81.47	-0.5807	0.2950	23.27	38.29	98.14	-0.9205	0.3223
51	24.47	40.34	81.54	-0.5735	0.2955	23.34	38.41	99.17	-0.9283	0.3230
52	24.46	40.40	81.61	-0.5664	0.2959	23.41	38.52	100.23	-0.9362	0.3237
53	24.46	40.45	81.67	-0.5593	0.2964	23.48	38.64	101.30	-0.9440	0.3244
54	24.46	40.51	81.74	-0.5522	0.2969	23.56	38.76	102.39	-0.9518	0.3251
55	24.46	40.57	81.80	-0.5451	0.2974	23.63	38.88	103.50	-0.9597	0.3258
56	24.46	40.63	81.87	-0.5380	0.2978	23.70	39.00	104.63	-0.9675	0.3265
57	24.45	40.69	81.94	-0.5309	0.2983	23.77	39.12	105.77	-0.9754	0.3272
58	24.45	40.75	82.00	-0.5238	0.2988	23.84	39.24	106.93	-0.9832	0.3279

59	24.45	40.80	82.07	-0.5167	0.2993	23.91	39.35	108.11	-0.9910	0.3287
60	24.44	40.86	82.14	-0.5096	0.2997	23.98	39.47	109.30	-0.9989	0.3294
61	24.44	40.92	82.20	-0.5024	0.3002	24.05	39.59	110.51	-1.0067	0.3301
62	24.43	40.98	82.27	-0.4953	0.3007	24.12	39.71	111.74	-1.0146	0.3308
63	24.43	41.04	82.34	-0.4882	0.3012	24.20	39.83	112.98	-1.0224	0.3315
64	24.42	41.10	82.40	-0.4811	0.3017	24.27	39.95	114.23	-1.0302	0.3323
65	24.42	41.15	82.47	-0.4740	0.3021	24.34	40.07	115.50	-1.0381	0.3330
66	24.41	41.21	82.54	-0.4669	0.3026	24.41	40.19	116.79	-1.0459	0.3337
67	24.41	41.27	82.60	-0.4598	0.3031	24.48	40.30	118.08	-1.0538	0.3344
68	24.40	41.33	82.67	-0.4527	0.3036	24.55	40.42	119.39	-1.0616	0.3352
69	24.40	41.39	82.74	-0.4456	0.3041	24.62	40.54	120.71	-1.0694	0.3359
70	24.39	41.45	82.80	-0.4385	0.3046	24.69	40.66	122.04	-1.0773	0.3366

To calculate the age-related z-score for plasma intact FGF23, the determined concentration x together with the corresponding age-related values for L, M, and S are used according to the formula $z = [(x/M)^L-1]/S×L$. L, skewness; M, median; S, coefficient of variation.

Supplementary Table S7. Age-specific percentile limits and LMS vales for plasma total FGF23 (RU/mL) in men and women

Age (yrs)	Men					
	2.5th	*50th (M)*	*97.5th*	*L*	*M (50th)*	*S*
18-70	39.1	61.3	122.3	-0.7596	61.3	0.2743
	Women					
	2.5th	*50th*	*97.5th*	*L*	*M*	*S*
18-70	40.2	73.6	223.6	-4.0993	179.3526	0.2113

To calculate the age-related z-score for plasma total FGF23 in males, the determined concentration x together with the corresponding age-related values for L, M, and S are used according to the formula $z = [(x/M)^L-1]/S\times L$. Before computing the age-adjusted z-score for total FGF23 in females, 100 must be added to the measured concentration x, due to mathematical requirements of the RefCurv software. The formula that should be used for females is $z = [((x+100)/M)^L-1]/SxL$. L, skewness; M, median; S, coefficient of variation.

Supplementary Table S8. Age-specific percentile limits and LMS values for plasma sKlotho (pg/mL) in men and women

Age (yrs)	Men					Women				
	2.5th	*50th (M)*	*97.5th*	*L*	*S*	*2.5th*	*50th (M)*	*97.5th*	*L*	*S*
18	427	955	1837	0.2874	0.3671	523	1226	2684	0.1004	0.4161
19	425	948	1822	0.2840	0.3664	515	1207	2637	0.1043	0.4155
20	423	941	1808	0.2806	0.3657	508	1189	2591	0.1081	0.4148
21	421	934	1794	0.2773	0.3650	500	1170	2545	0.1120	0.4142
22	419	927	1779	0.2739	0.3644	492	1152	2500	0.1158	0.4135
23	417	920	1765	0.2706	0.3637	484	1134	2455	0.1196	0.4129
24	415	913	1751	0.2672	0.3630	476	1116	2410	0.1235	0.4123
25	412	906	1736	0.2638	0.3624	469	1098	2366	0.1273	0.4116
26	410	898	1722	0.2605	0.3617	461	1080	2323	0.1311	0.4110
27	408	891	1708	0.2571	0.3610	453	1062	2280	0.1350	0.4104
28	406	884	1693	0.2538	0.3604	446	1045	2237	0.1388	0.4097
29	404	877	1679	0.2504	0.3597	438	1027	2195	0.1427	0.4091
30	402	870	1665	0.2470	0.3590	431	1010	2154	0.1465	0.4085
31	399	863	1650	0.2437	0.3584	424	993	2113	0.1503	0.4078
32	397	856	1636	0.2403	0.3577	417	977	2073	0.1542	0.4072
33	395	849	1622	0.2369	0.3570	410	960	2034	0.1580	0.4066
34	393	842	1608	0.2336	0.3564	403	944	1996	0.1619	0.4059
35	390	835	1593	0.2302	0.3557	396	929	1958	0.1657	0.4053
36	388	828	1579	0.2269	0.3551	390	913	1922	0.1695	0.4047
37	386	821	1565	0.2235	0.3544	383	899	1887	0.1734	0.4041
38	383	813	1551	0.2201	0.3538	377	885	1854	0.1772	0.4034
39	381	806	1537	0.2168	0.3531	372	871	1822	0.1811	0.4028
40	379	799	1522	0.2134	0.3525	366	858	1791	0.1849	0.4022
41	376	792	1508	0.2101	0.3518	361	846	1762	0.1887	0.4016
42	374	785	1494	0.2067	0.3512	356	834	1734	0.1926	0.4009
43	371	778	1480	0.2033	0.3505	351	823	1708	0.1964	0.4003
44	369	771	1466	0.2000	0.3499	347	813	1683	0.2003	0.3997
45	366	764	1452	0.1966	0.3492	342	803	1659	0.2041	0.3991
46	364	757	1438	0.1933	0.3486	339	794	1637	0.2079	0.3985
47	361	750	1423	0.1899	0.3479	335	785	1615	0.2118	0.3979
48	359	743	1409	0.1865	0.3473	331	777	1595	0.2156	0.3972
49	356	735	1395	0.1832	0.3466	328	769	1576	0.2195	0.3966
50	353	728	1381	0.1798	0.3460	325	761	1557	0.2233	0.3960
51	351	721	1367	0.1765	0.3454	322	754	1540	0.2271	0.3954
52	348	714	1353	0.1731	0.3447	319	748	1523	0.2310	0.3948
53	346	707	1339	0.1697	0.3441	316	741	1508	0.2348	0.3942
54	343	700	1325	0.1664	0.3435	314	735	1493	0.2387	0.3936
55	340	693	1311	0.1630	0.3428	311	730	1479	0.2425	0.3930
56	337	686	1297	0.1597	0.3422	309	725	1465	0.2463	0.3924
57	335	679	1283	0.1563	0.3416	307	720	1453	0.2502	0.3917
58	332	672	1269	0.1529	0.3409	305	715	1441	0.2540	0.3911

59	330	665	1255	0.1496	0.3403	303	711	1429	0.2579	0.3905
60	326	658	1241	0.1462	0.3397	301	707	1418	0.2617	0.3899
61	324	650	1227	0.1429	0.3391	300	703	1408	0.2655	0.3893
62	321	643	1213	0.1395	0.3384	298	699	1398	0.2694	0.3887
63	318	636	1199	0.1361	0.3378	296	695	1388	0.2732	0.3881
64	315	629	1185	0.1328	0.3372	295	692	1378	0.2770	0.3875
65	312	622	1171	0.1294	0.3366	293	688	1369	0.2809	0.3869
66	309	615	1158	0.1260	0.3359	292	685	1359	0.2847	0.3863
67	306	608	1144	0.1227	0.3353	290	681	1349	0.2886	0.3857
68	303	601	1130	0.1193	0.3347	289	677	1340	0.2924	0.3851
69	301	594	1116	0.1160	0.3341	287	674	1330	0.2962	0.3845
70	298	587	1102	0.1126	0.3335	286	670	1320	0.3001	0.3839

To calculate the age-related z-score for plasma sKlotho, the determined concentration x together with the corresponding age and sex-related values for L, M, and S are used according to the formula $z = [(x/M)^L-1]/S\times L$. L, skewness; M, median; S, coefficient of variation.

Supplementary Table S9. Age-specific percentile limits and LMS values for the ratio of iFGF23/Pi (pg/μmol) in men and women

Age (yrs)	Men					Women				
	2.5th	*50th (M)*	*97.5th*	*L*	*S*	*2.5th*	*50th (M)*	*97.5th*	*L*	*S*
18	21.66	34.36	68.18	-0.6989	0.2778	20.26	31.06	61.64	-0.8647	0.2639
19	21.68	34.51	68.78	-0.6933	0.2797	20.15	31.14	62.94	-0.8593	0.2694
20	21.71	34.66	69.38	-0.6877	0.2816	20.04	31.22	64.31	-0.8540	0.2751
21	21.73	34.81	69.99	-0.6820	0.2835	19.93	31.31	65.74	-0.8486	0.2809
22	21.76	34.96	70.61	-0.6764	0.2854	19.81	31.39	67.25	-0.8433	0.2869
23	21.78	35.11	71.23	-0.6708	0.2873	19.70	31.47	68.84	-0.8379	0.2929
24	21.80	35.26	71.85	-0.6652	0.2893	19.58	31.56	70.51	-0.8325	0.2991
25	21.82	35.42	72.48	-0.6595	0.2912	19.46	31.64	72.25	-0.8272	0.3054
26	21.84	35.57	73.12	-0.6539	0.2932	19.34	31.72	74.07	-0.8218	0.3117
27	21.85	35.72	73.76	-0.6483	0.2952	19.22	31.81	75.96	-0.8165	0.3181
28	21.87	35.87	74.41	-0.6427	0.2972	19.10	31.89	77.91	-0.8111	0.3244
29	21.88	36.02	75.06	-0.6370	0.2992	18.98	31.97	79.91	-0.8058	0.3308
30	21.90	36.17	75.72	-0.6314	0.3012	18.86	32.06	81.95	-0.8004	0.3370
31	21.91	36.32	76.38	-0.6258	0.3033	18.75	32.14	83.99	-0.7950	0.3431
32	21.92	36.47	77.05	-0.6202	0.3053	18.65	32.22	86.01	-0.7897	0.3490
33	21.94	36.62	77.72	-0.6145	0.3074	18.55	32.31	87.98	-0.7843	0.3545
34	21.95	36.77	78.40	-0.6089	0.3095	18.46	32.39	89.87	-0.7790	0.3597
35	21.96	36.93	79.09	-0.6033	0.3116	18.38	32.47	91.65	-0.7736	0.3646
36	21.96	37.08	79.78	-0.5977	0.3137	18.31	32.56	93.32	-0.7683	0.3691
37	21.97	37.23	80.48	-0.5920	0.3158	18.25	32.64	94.87	-0.7629	0.3732
38	21.98	37.38	81.18	-0.5864	0.3179	18.20	32.72	96.28	-0.7575	0.3769
39	21.98	37.53	81.89	-0.5808	0.3201	18.15	32.80	97.55	-0.7522	0.3802
40	21.98	37.68	82.60	-0.5752	0.3223	18.12	32.89	98.68	-0.7468	0.3832
41	21.99	37.83	83.32	-0.5695	0.3244	18.09	32.97	99.63	-0.7415	0.3858
42	21.99	37.98	84.04	-0.5639	0.3266	18.07	33.05	100.40	-0.7361	0.3879
43	21.99	38.13	84.77	-0.5583	0.3288	18.06	33.14	100.95	-0.7308	0.3896
44	21.99	38.28	85.51	-0.5527	0.3311	18.07	33.22	101.29	-0.7254	0.3907
45	21.99	38.44	86.25	-0.5470	0.3333	18.08	33.30	101.38	-0.7200	0.3914
46	21.98	38.59	87.00	-0.5414	0.3356	18.11	33.39	101.26	-0.7147	0.3914
47	21.98	38.74	87.76	-0.5358	0.3378	18.15	33.47	100.91	-0.7093	0.3910
48	21.97	38.89	88.52	-0.5302	0.3401	18.20	33.55	100.39	-0.7040	0.3901
49	21.97	39.04	89.28	-0.5245	0.3424	18.26	33.64	99.70	-0.6986	0.3889
50	21.96	39.19	90.06	-0.5189	0.3447	18.32	33.72	98.90	-0.6933	0.3872
51	21.95	39.34	90.84	-0.5133	0.3471	18.40	33.80	98.01	-0.6879	0.3854
52	21.94	39.49	91.62	-0.5077	0.3494	18.48	33.89	97.05	-0.6825	0.3832
53	21.93	39.64	92.41	-0.5020	0.3518	18.56	33.97	96.06	-0.6772	0.3809
54	21.92	39.79	93.21	-0.4964	0.3542	18.65	34.05	95.04	-0.6718	0.3785
55	21.90	39.95	94.01	-0.4908	0.3566	18.74	34.13	94.02	-0.6665	0.3760
56	21.89	40.10	94.82	-0.4852	0.3590	18.84	34.22	93.00	-0.6611	0.3734
57	21.87	40.25	95.64	-0.4795	0.3614	18.93	34.30	92.01	-0.6558	0.3707
58	21.85	40.40	96.46	-0.4739	0.3639	19.03	34.38	91.05	-0.6504	0.3681

59	21.84	40.55	97.28	-0.4683	0.3663	19.13	34.47	90.13	-0.6450	0.3655
60	21.82	40.70	98.12	-0.4627	0.3688	19.22	34.55	89.26	-0.6397	0.3630
61	21.80	40.85	98.96	-0.4570	0.3713	19.32	34.63	88.42	-0.6343	0.3605
62	21.77	41.00	99.80	-0.4514	0.3738	19.41	34.72	87.63	-0.6290	0.3581
63	21.75	41.15	100.66	-0.4458	0.3763	19.51	34.80	86.86	-0.6236	0.3557
64	21.72	41.30	101.52	-0.4401	0.3789	19.60	34.88	86.11	-0.6183	0.3532
65	21.70	41.46	102.38	-0.4345	0.3815	19.70	34.97	85.38	-0.6129	0.3508
66	21.67	41.61	103.25	-0.4289	0.3840	19.80	35.05	84.66	-0.6075	0.3483
67	21.64	41.76	104.13	-0.4233	0.3866	19.90	35.13	83.95	-0.6022	0.3458
68	21.61	41.91	105.01	-0.4176	0.3893	20.00	35.21	83.26	-0.5968	0.3433
69	21.58	42.06	105.90	-0.4120	0.3919	20.10	35.30	82.58	-0.5915	0.3408
70	21.55	42.21	106.80	-0.4064	0.3945	20.21	35.38	81.92	-0.5861	0.3383

To calculate the age-related z-score for the ratio of iFGF23/Pi, the determined concentration x together with the corresponding age and sex-related values for L, M, and S are used according to the formula $z = [(x/M)^L - 1]/S \times L$. L, skewness; M, median; S, coefficient of variation.

Supplementary Table S10. Age-specific percentile limits and LMS values for the ratio of iFGF23/sKlotho (pg/pg) in men and women

Age (yrs)	Men					Women				
	2.5th	*50th (M)*	*97.5th*	*L*	*S*	*2.5th*	*50th (M)*	*97.5th*	*L*	*S*
18	0.0160	0.0404	0.1017	0.0062	0.4722	0.0103	0.0278	0.0930	-0.1746	0.5548
19	0.0162	0.0409	0.1034	0.0010	0.4727	0.0106	0.0286	0.0948	-0.1703	0.5530
20	0.0164	0.0415	0.1051	-0.0042	0.4733	0.0109	0.0294	0.0967	-0.1660	0.5513
21	0.0167	0.0420	0.1068	-0.0094	0.4738	0.0112	0.0302	0.0985	-0.1617	0.5495
22	0.0169	0.0426	0.1085	-0.0146	0.4744	0.0115	0.0309	0.1003	-0.1574	0.5477
23	0.0171	0.0431	0.1103	-0.0198	0.4749	0.0118	0.0317	0.1020	-0.1531	0.5460
24	0.0174	0.0436	0.1120	-0.0250	0.4755	0.0121	0.0325	0.1038	-0.1488	0.5442
25	0.0176	0.0442	0.1138	-0.0302	0.4760	0.0124	0.0333	0.1055	-0.1445	0.5425
26	0.0178	0.0447	0.1156	-0.0354	0.4766	0.0127	0.0340	0.1072	-0.1402	0.5408
27	0.0181	0.0452	0.1174	-0.0406	0.4771	0.0130	0.0348	0.1089	-0.1358	0.5390
28	0.0183	0.0458	0.1192	-0.0458	0.4777	0.0133	0.0356	0.1105	-0.1315	0.5373
29	0.0185	0.0463	0.1211	-0.0510	0.4783	0.0136	0.0364	0.1122	-0.1272	0.5356
30	0.0188	0.0469	0.1229	-0.0561	0.4788	0.0139	0.0371	0.1138	-0.1229	0.5339
31	0.0190	0.0474	0.1248	-0.0613	0.4794	0.0142	0.0379	0.1154	-0.1186	0.5322
32	0.0193	0.0479	0.1266	-0.0665	0.4799	0.0145	0.0387	0.1170	-0.1143	0.5305
33	0.0195	0.0485	0.1285	-0.0717	0.4805	0.0148	0.0394	0.1185	-0.1100	0.5288
34	0.0197	0.0490	0.1305	-0.0769	0.4811	0.0151	0.0402	0.1201	-0.1057	0.5271
35	0.0200	0.0496	0.1324	-0.0821	0.4816	0.0154	0.0410	0.1216	-0.1014	0.5254
36	0.0202	0.0501	0.1343	-0.0873	0.4822	0.0157	0.0418	0.1232	-0.0971	0.5237
37	0.0204	0.0506	0.1363	-0.0925	0.4828	0.0160	0.0425	0.1247	-0.0928	0.5221
38	0.0207	0.0512	0.1383	-0.0977	0.4833	0.0163	0.0433	0.1261	-0.0885	0.5204
39	0.0209	0.0517	0.1403	-0.1029	0.4839	0.0166	0.0441	0.1276	-0.0842	0.5187
40	0.0212	0.0523	0.1423	-0.1081	0.4845	0.0169	0.0449	0.1291	-0.0799	0.5171
41	0.0214	0.0528	0.1443	-0.1133	0.4850	0.0172	0.0456	0.1305	-0.0755	0.5154
42	0.0216	0.0533	0.1464	-0.1185	0.4856	0.0175	0.0464	0.1320	-0.0712	0.5138
43	0.0219	0.0539	0.1485	-0.1237	0.4862	0.0179	0.0472	0.1334	-0.0669	0.5121
44	0.0221	0.0544	0.1506	-0.1289	0.4867	0.0182	0.0480	0.1348	-0.0626	0.5105
45	0.0224	0.0549	0.1527	-0.1341	0.4873	0.0185	0.0487	0.1362	-0.0583	0.5089
46	0.0226	0.0555	0.1548	-0.1393	0.4879	0.0188	0.0495	0.1375	-0.0540	0.5072
47	0.0229	0.0560	0.1570	-0.1445	0.4884	0.0191	0.0503	0.1389	-0.0497	0.5056
48	0.0231	0.0566	0.1592	-0.1497	0.4890	0.0194	0.0511	0.1403	-0.0454	0.5040
49	0.0233	0.0571	0.1614	-0.1549	0.4896	0.0197	0.0518	0.1416	-0.0411	0.5024
50	0.0236	0.0576	0.1636	-0.1601	0.4902	0.0201	0.0526	0.1429	-0.0368	0.5008
51	0.0238	0.0582	0.1658	-0.1653	0.4907	0.0204	0.0534	0.1443	-0.0325	0.4992
52	0.0241	0.0587	0.1681	-0.1705	0.4913	0.0207	0.0541	0.1456	-0.0282	0.4976
53	0.0243	0.0593	0.1704	-0.1757	0.4919	0.0210	0.0549	0.1469	-0.0239	0.4960
54	0.0246	0.0598	0.1727	-0.1809	0.4925	0.0213	0.0557	0.1482	-0.0196	0.4944
55	0.0248	0.0603	0.1751	-0.1861	0.4930	0.0216	0.0565	0.1494	-0.0152	0.4928
56	0.0251	0.0609	0.1774	-0.1913	0.4936	0.0220	0.0572	0.1507	-0.0109	0.4913
57	0.0253	0.0614	0.1798	-0.1965	0.4942	0.0223	0.0580	0.1520	-0.0066	0.4897

58	0.0255	0.0619	0.1822	-0.2017	0.4948	0.0226	0.0588	0.1532	-0.0023	0.4881
59	0.0258	0.0625	0.1847	-0.2069	0.4953	0.0229	0.0596	0.1544	0.0020	0.4866
60	0.0260	0.0630	0.1872	-0.2121	0.4959	0.0233	0.0603	0.1557	0.0063	0.4850
61	0.0263	0.0636	0.1897	-0.2173	0.4965	0.0236	0.0611	0.1569	0.0106	0.4835
62	0.0265	0.0641	0.1922	-0.2225	0.4971	0.0239	0.0619	0.1581	0.0149	0.4819
63	0.0268	0.0646	0.1948	-0.2277	0.4977	0.0242	0.0627	0.1593	0.0192	0.4804
64	0.0270	0.0652	0.1974	-0.2328	0.4983	0.0246	0.0634	0.1605	0.0235	0.4788
65	0.0273	0.0657	0.2000	-0.2380	0.4988	0.0249	0.0642	0.1617	0.0278	0.4773
66	0.0275	0.0663	0.2027	-0.2432	0.4994	0.0252	0.0650	0.1629	0.0321	0.4758
67	0.0278	0.0668	0.2054	-0.2484	0.5000	0.0255	0.0658	0.1640	0.0364	0.4743
68	0.0280	0.0673	0.2081	-0.2536	0.5006	0.0259	0.0665	0.1652	0.0407	0.4727
69	0.0283	0.0679	0.2108	-0.2588	0.5012	0.0262	0.0673	0.1664	0.0451	0.4712
70	0.0285	0.0684	0.2136	-0.2640	0.5018	0.0265	0.0681	0.1675	0.0494	0.4697

To calculate the age-related z-score for the ratio of iFGF23/sKlotho, the determined concentration x together with the corresponding age and sex-related values for L, M, and S are used according to the formula $z = [(x/M)^L - 1]/S \times L$. L, skewness; M, median; S, coefficient of variation.
